# Sympatric Occurrence of Five Exophilic Tick Species in the Levice Region (Southwestern Slovakia) and Their Infection with Tick-Borne Pathogens

**DOI:** 10.3390/pathogens15040382

**Published:** 2026-04-02

**Authors:** Slávka Purgatová, Barbara Mangová, Diana Selyemová, Zuzana Krumpálová, Michal Chvostáč, Yuliya M. Didyk, Petra Rajská, Mária Kazimírová, Veronika Rusňáková Tarageľová

**Affiliations:** 1 Institute of Zoology, Slovak Academy of Sciences, 84506 Bratislava, Slovakia; purgatova8@gmail.com (S.P.); mangova.barbara@gmail.com (B.M.); diana.selyemova@gmail.com (D.S.); michal.chvostac@gmail.com (M.C.); yumdidyk@gmail.com (Y.M.D.); rajskapetra7@gmail.com (P.R.); 2 Department of Ecology and Environmental Studies, Faculty of Natural Sciences and Informatics, Constantine the Philosopher University, 94901 Nitra, Slovakia; zkrumpalova@ukf.sk; 3 I.I. Schmalhausen Institute of Zoology NAS of Ukraine, 01054 Kyiv, Ukraine

**Keywords:** ticks, pathogens, wildlife, reservoir host, urban site, rural site, natural site

## Abstract

Among the 24 tick species documented in Slovakia, *Ixodes ricinus* is the most widespread and abundant. In some sites, 2–5 epidemiologically important tick species co-occur. Such sites represent hotspots for studying the co-circulation of tick-borne pathogens. Sympatric occurrence of five exophilic species (*I. ricinus*, *Dermacentor reticulatus*, *D. marginatus*, *Haemaphysalis concinna*, and *H. inermis*) was confirmed in the environs of the Žemberovce village (Levice region, south-western Slovakia). Here, the seasonal activity and abundance of questing ticks and the tick infestation of game and sheep were investigated. Questing ticks, spleens of game, and ticks removed from game and sheep were examined for the presence of tick-borne pathogens (*Babesia* spp., *Theileria* spp., *Anaplasma phagocytophilum*, *Rickettsia* spp., *Borrelia burgdorferi* s.l., and *Borrelia miyamotoi*) by molecular methods. Among the questing ticks, *I. ricinus* prevailed. Presence of *Babesia crassa*, *B. microti*, *Rickettsia helvetica*, *R. raoultii*, *A. phagocytophilum*, *Borrelia afzelii*, *B. garinii*, *B. valaisiana*, *B. burgdorferi* sensu stricto and *B. miyamotoi* was detected. *Dermacentor marginatus*, *I. ricinus* and *H. concinna* were collected from sheep. They were infected with *A. phagocytophilum*, *A. ovis*, *R. slovaca*, and *R. raoultii*. *Anaplasma phagocytophilum* was detected in all examined red deer and roe deer and in 55.6% of wild boar. All cervids were positive for *Theileria* spp. Infestation of game with all five tick species, with a predominance of *I. ricinus*, was confirmed. In these ticks, *A. phagocytophilum*, *R. helvetica*, *R. raoultii*, *Babesia* sp. hc-hlj212, *B. crassa*, *B. microti*, *Babesia* spp. and *B. miyamotoi* were detected. This study confirmed the presence of *B. crassa* in Slovakia for the first time. The investigated area, with the co-occurrence of five exophilic tick species and a wide spectrum of tick-borne pathogens, represents an epidemiologically important hotspot with the risk of infections of humans and domestic animals.

## 1. Introduction

Ticks are significant vectors of pathogens of medical and veterinary importance. In Central Europe, including Slovakia, climatic changes, alterations in land use patterns, and the abundance and distribution of wildlife hosts resulted in the expansion, increase in quantity, as well as in the prolonged seasonal activity of the most medically significant tick species *Ixodes ricinus* and *Dermacentor reticulatus* [1,2,3,4,5,6,7]. The changing spatial and temporal distribution of these tick species is also connected with increased prevalence and diversity of the tick-borne pathogens they transmit [8,9,10].

Sympatric occurrence of multiple tick species within the same habitats is regarded as a critical but understudied phenomenon in tick ecology and epidemiology of tick-borne diseases, which, however, affects the emergence and spread of tick-borne pathogens [11].

Changing land use patterns and habitat fragmentation create more contact zones for tick species with different requirements to microclimatic conditions, which, together with the presence of diverse vertebrate host communities (small mammals, birds, and ungulates), support the existence of overlapping tick populations and may promote co-feeding transmission of pathogens between more tick species feeding on the same host [12]. This, in turn, can result in an increased diversity of pathogens in habitats with sympatric occurrence of more tick species [13]. Understanding the conditions under which multiple tick species and their associated pathogens co-occur is essential for predicting infection risk and for designing effective surveillance and control strategies. However, most studies focus on associations between single pathogens and single tick species, while studies on complex tick-host–pathogen interactions in a system with sympatric occurrence of ticks are relatively rare.

In many locations of Central Europe, co-occurrence of *I. ricinus* and *D. reticulatus* has been reported [14,15,16]. In some areas, depending on the habitat type, the less abundant *Haemaphysalis concinna* with patchy distribution co-exists with both of the above species [17,18,19], or occurs in sympatry only with *I. ricinus* [20,21]. Reports on sympatric occurrence of *D. reticulatus* and *Dermacentor marginatus* [22], of *I. ricinus*, *D. marginatus* and *D. reticulatus* [23], or of *I. ricinus*, *H. concinna* and *Haemaphysalis inermis* [24] in connection with pathogen detections are relatively rare. Information on the co-occurrence of four to five epidemiologically important tick species has been published, e.g., recently from Hungary [25] and Croatia [26]; however, investigations that involve also tick–host–pathogen interactions have been carried out only in a few locations, including a site in eastern Slovakia [27,28].

By combining ecological and molecular biology approaches, our study aims to contribute to the understanding of multi-tick and multi-pathogen systems based on an example of a rural area in south-western Slovakia.

The specific objectives of the study were: (1) to determine the seasonal occurrence of ticks and their infection with tick-borne pathogens in an area in south-western Slovakia where five exophilic tick species of medical and veterinary importance co-occur; (2) to investigate the role of wild ungulates as host of tick-borne pathogens and ticks in the study area; and (3) to determine the genetic diversity of the detected pathogens with focus on strains pathogenic to humans and domestic animals.

## 2. Materials and Methods

### 2.1. Characteristics of the Study Area

The village of Žemberovce (48°15′30″ N, 18°44′30″ E) is located in western Slovakia, at an altitude of 215 m a.s.l., at the junction of the Carpathians and the Pannonian Basin. The majority of the territory forms part of the Pannonian Basin, as it belongs to the Danubian Lowland region and, within it, to the Danubian Upland. This unit is further subdivided into subunits and parts. The municipality and its surroundings are located in the Ipeľská Pahorkatina Upland subunit, specifically in the Brhlovské Podhorie Foothills and the Bátovská Pahorkatina Upland. A smaller part of the cadastral area belongs to the Štiavnické Mountains, within the Sitnianska Vrchovina Highlands subunit, specifically the Sitnianske Predhorie Foothills. These units are already classified within the Slovak Central Mountains, which are part of the Western Carpathians. Both xerothermic and humid habitats occur in this area. Thermophilous forest communities in the surroundings of the village are represented mainly by Carpathian oak–hornbeam forests, while on suitable sites, oak–Turkey oak forests can also be found. The area hosts numerous animal species associated with forest habitats; however, a high proportion of species is also linked to watercourses and their riparian vegetation, as well as to meadows, orchards, gardens, fields, and their margins.

**Figure 1:**
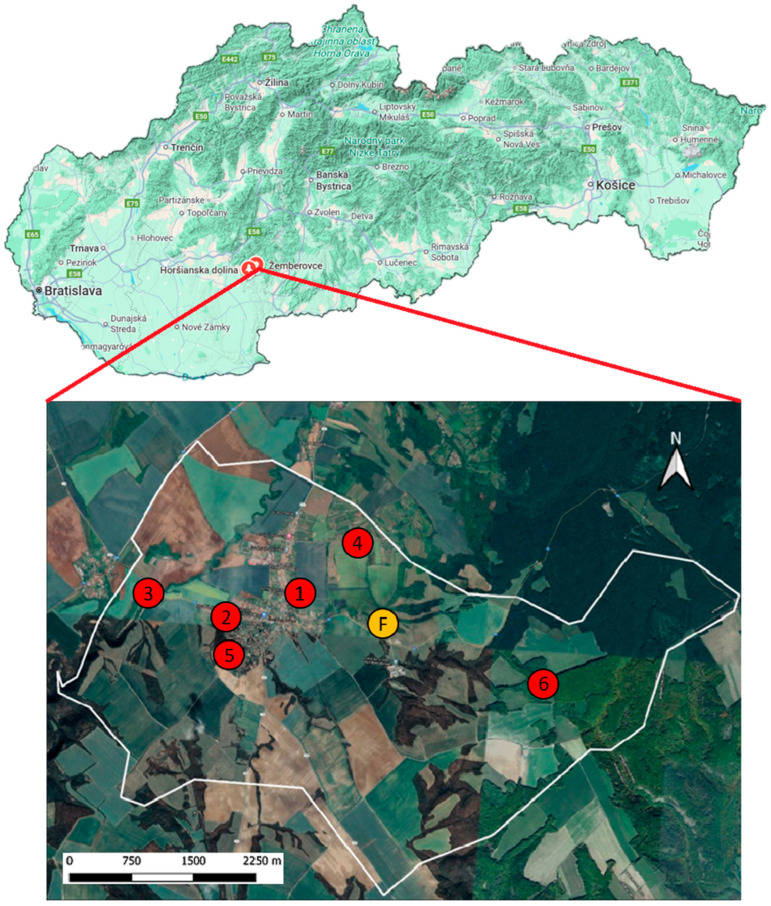
Location of the village of Žemberovce and Horšianska Dolina National Nature Reserve in the map of Slovakia (**upper panel**; created with Google Maps for Windows 10). Map of Žemberovce village with study sites 1–6: 1—cemetery, 2—park, 3—at the Sikenica River, 4—vineyard, 5—meadow, 6—hunter’s cottage, and F—Selec farm (**lower panel**; created with QGIS 3.16).

The study sites (1–6) were selected to differ in their natural conditions. In Žemberovce, two study sites were located within the residential area (1—cemetery, 2—park), and four study sites were situated in the rural zone (3—at the Sikenica River, 4—vineyard, 5—meadow, 6—hunter’s cottage) (Figure 1). For site comparison, a location in the Horšianska Dolina National Nature Reserve (site 7), at a distance of approximately 7 km from the village, was chosen (Figure 1) [29].

### 2.2. Biological Samples

Questing ticks were collected from the vegetation in monthly intervals by flagging [30] at the seven study sites during 2021–2022. In each site, 3 × 10 m long transects were chosen, and flagging was carried out using a 1 × 1 m white blanket.

In cooperation with the local hunting association in Žemberovce, feeding ticks and spleen samples were collected from hunted game animals in 2021 and 2022. The game animals were killed for hunting reasons during the legal hunting seasons and not specifically for this study. Therefore, approval of the ethics committee was not required. The hunted animals comprised red deer (*Cervus elaphus*), roe deer (*Capreolus capreolus*), and wild boar (*Sus scrofa*). In addition, feeding ticks were collected from sheep at the Selec farm (belonging to the agricultural cooperative in Žemberovce) in May 2022. The ticks were removed by the first author of the paper (S. Purgatová), with the oral consent of the owner of the farm.

Collected spleen samples and ticks were stored in 70% ethanol at 4 °C until further molecular analyses. Ticks were identified to species, developmental stage, and sex under a stereomicroscope by using standard identification keys [31,32].

### 2.3. DNA Isolation

Genomic DNA was isolated individually from questing ticks by the method of alkaline hydrolysis with modifications [33] and from feeding ticks and spleen samples using the commercial Macherey-Nagel NucleoSpin^®^ Tissue kit (Düren, Germany), in accordance with the manufacturer’s protocol. Quantity and quality of the isolated DNA were measured with a Nanodrop 2000c spectrophotometer (Thermo Fisher Scientific, Wilmington, DE, USA), and the DNA samples were stored at −20 °C until further analyses.

### 2.4. PCR Detection of Tick-Borne Pathogens

The DNA samples from ticks and spleen were screened for the presence of *Babesia* spp. and *Theileria* spp. (Piroplasmida), *Anaplasma phagocytophilum*, *Rickettsia* spp., *Borrelia burgdorferi* sensu lato (s.l.), and *Borrelia miyamotoi* by PCR-based methods. Ticks collected from sheep were also tested for the presence of *Anaplasma ovis*.

Piroplasmid DNA amplification by PCR was carried out following the protocol described by Casati et al. [34]; genus-specific BJ1 and BN2 primers were used to amplify a 450 bp region of the 18S rRNA gene.

*Anaplasma phagocytophilum* DNA was detected using a real-time PCR; the *msp*2 gene was amplified. Primers ApMSP2f and ApMSP2r [35] were used to generate a 77 bp fragment by using the ApMSP2p-FAM TaqMan probe. Selected positive samples from the spleens of game animals were further tested by nested PCR targeting a 344 bp fragment of the *msp*4 gene [36] and by touch-down PCR targeting a 530 bp fragment of the *groEL* gene [37] to identify *A. phagocytophilum* strains by sequencing.

Molecular detection of *Rickettsia* spp. was performed by PCR with primers D767f and D1390r, amplifying a 626 bp fragment of the *sca*4 gene [38].

A PCR assay targeting a 75 bp fragment of the 23S rRNA gene, using primers Bb23Sr and Bb2Sf, was used for the detection of *B. burgdorferi* s.l. All *Borrelia*-positive samples were further tested by conventional PCR. IgSA and IgSB primers were used to amplify a 230 bp portion of the 5S–23S (rrfA-rrlB) rRNA intergenic spacer [39].

*Borrelia miyamotoi* was detected using a real-time PCR to amplify a 1256 bp fragment of 16 S rRNA gene. Bmp41f and Bmp41r primers and Bmp41 probe were used [40].

PCR for detection of *Anaplasma ovis* was performed according to the protocol described by de la Fuente et al. [41], using species-specific MSP43/MSP45 primers targeting an 854 bp fragment of the *msp*4 gene.

Negative (nuclease-free water) and positive controls (DNA from ticks positive for *Theileria capreoli*, *A. phagocytophilum*, *B. valaisiana*, *B. miyamotoi*, and *R. slovaca*, respectively) were used in all PCR reactions. Primers and probes used in the PCR reactions are listed in Table 1. Detailed descriptions of the PCR protocols are provided in Supplementary File S1.

Amplicons derived from samples positive for *B. burgdorferi* s.l., *Rickettsia* spp. and *Babesia*/*Theileria* from questing ticks; for *Babesia*/*Theileria* from spleens; for *Rickettsia* spp. and *A. ovis* from ticks feeding on sheep; for *Rickettsia* spp. from ticks feeding on game; and amplicons from randomly selected samples positive for *Babesia*/*Theileria* from ticks feeding on game, as well as amplicons of samples positive for the *A. phagocytophilum groEL* and *msp4* genes from spleen, were purified using the QIAquick Spin PCR Purification Kit (Qiagen, Hilden, Germany) according to the instructions of the manufacturer. The amplicons were analyzed by sequencing with forward and reverse primers employed for PCR amplification. Sequencing was carried out by Eurofins Genomics Europe (www.eurofinsgenomics.eu). Unique sequences for Slovakia, obtained from DNA isolates from ticks, questing and feeding on game and sheep, and from the spleens of cervids and wild boar, were deposited in the GenBank database (see Supplementary Table S1). They included an 18S rRNA sequence of *Babesia crassa* (Access. No. PX992076), *sca*4 sequences of two *Ricketsia raoultii* genotypes (Access. No. PX991675, PX991676), one *Rickettsia helvetica* (Access. No. PX991677), one *Rickettsia slovaca* (Access. No. PX991678), 12 *groEL* (Access. No. PZ171433-PZ171444) and six *msp*4 (Access No. PZ171427-PZ171432) sequences of *A. phagocytophilum*, two *msp*4 sequences of *Anaplasma ovis* (Access. No. PX991679, PX991680), seventeen 5S–23S (rrfA-rrlB) rRNA sequences of *B. burgdorferi* s.l.: nine *B. afzelii* (Access. No. PZ171410-PZ171418), four *B. garinii* (Access. No. PZ171419-PZ171422), three *B. valaisiana* (Access No. PZ171423-PZ171425) and one *B. burgdorferi* s.s. (Access. No. PZ171426). The other sequences generated in this study are available at https://doi.org/10.6084/m9.figshare.31869478.

### 2.5. Phylogenetic Analyses

The obtained nucleotide sequences were manually edited using the MEGA7.0 software [42]. The determined 18S rRNA gene nucleotide sequences of *Babesia*/*Theileria* species, the *msp4* and *groEL* sequences of *A. phagocytophilum*, the *msp4* sequences of *A. ovis*, and the *sca4* gene sequences of *Rickettsia* species were used as queries in BLASTn+ 2.17.0 searches in order to identify and download the most closely related gene sequences of well-defined piroplasmid, *Anaplasma* and *Rickettsia* species from the GenBank database. Details on the source of the sequences obtained in this study that were included in phylogenetic analyses are available in Supplementary Table S1 and at https://doi.org/10.6084/m9.figshare.31869478.

Phylogenetic analyses were carried out using the MEGA7 software [42]. In the final datasets, there were 530, 339, 811, 582 and 471 positions for *A. phagocytophilum groEL*, *A. phagocytophilum msp*4, *A. ovis msp*4, *Rickettsia sca*4, and *Babesia*/*Theileria* 18S rRNA genes, respectively.

After estimation of the shape parameter, the evolutionary history was inferred by applying the Maximum Likelihood method based on the Tamura 3-parameter model for *A. phagocytophilum groEL* and *A. ovis msp*4 gene sequences, the Kimura 2-parameter model with Gamma distribution for *A. phagocytophilum msp*4 and the Tamura 3-parameter model with Gamma distribution for *Babesia*/*Theileria* 18S rRNA gene sequences. The Tamura 3-parameter model with invariant sites was applied for *Rickettsia sca*4 gene sequences. Models with the lowest Bayesian information criterion scores that are considered to describe substitution patterns the best were selected. Bootstrap values were estimated based on 1000 replicates. The percentage of trees in which the associated taxa clustered together is shown next to the branches.

### 2.6. Statistical Analyses

Ninety-five percent confidence intervals (95% CI) for each proportion were calculated by using the Wilson Score interval method [43] at the website http://epitools.ausvet.com.au.

Multivariate principal component analysis (PCA) implemented in the updated PAST 3 software package [44] was performed using sampling sites as statistical units (river, vineyards, meadow, hunter’s cottage, and Natural Reserve Horšianska Dolina). The variables included in the analysis were: the number of collected individuals of each tick species (*Ixodes ricinus*, *Haemaphysalis concinna*, *H. inermis*, *Dermacentor reticulatus*, and *D. marginatus*), and the prevalence (%) of detected pathogens (*Borrelia burgdorferi* s.l., *Borrelia miyamotoi*, *Rickettsia* spp., *Anaplasma phagocytophilum*, and *Theileria*/*Babesia* spp.) calculated for each site. PCA was performed using the correlation matrix, which standardizes variables to a zero mean and unit variance. This approach ensures comparability between tick counts and pathogen prevalence (%). The same approach was chosen for the evaluation of correlation between the feeding ticks on their preferred hosts—specimen number and pathogen prevalence in feeding ticks and animal tissue. The PCA was performed using source material as statistical units (wild boar ticks/spleens, roe deer ticks/spleens, red deer ticks/spleens, sheep ticks). The variables included in the analysis were: the number of collected individuals of each tick species (*I. ricinus*, *H. concinna*, *H. inermis*, *D. reticulatus*, and *D. marginatus*), and the prevalence (%) of detected pathogens (*B. miyamotoi*, *Rickettsia* spp., *Anaplasma* spp., and *Theileria*/*Babesia* spp.) calculated for each source. PCA was performed using the correlation matrix, which standardizes variables to a zero mean and unit variance. This approach ensures comparability between tick counts and pathogen prevalence (%).

## 3. Results

### 3.1. Questing Ticks

In total, 323 questing ticks were collected from five study sites. No ticks were found in the residential area of the village (sites 1 and 2). The collections included *Ixodes ricinus* (50.5%), *Haemaphysalis concinna* (29.1%), *Haemaphysalis inermis* (11.5%), *Dermacentor marginatus* (4.6%), and *Dermacentor reticulatus* (4.3%) (Table 2). The highest number of ticks (*n* = 113) was collected in the vineyard (site 4). Along with increasing abundance, the species diversity decreased towards the natural reserve (site 7). The highest species diversity was found close to a hunter’s cottage (site 6), where five tick species were found. Four species were recorded in the vineyard (site 4) in spite of a considerable anthropogenic disturbance. A higher diversity of tick species was recorded in human-altered sites (sites 4 and 6), including *H. concinna* and *H. inermis*, whereas *I. ricinus* predominated in less disturbed sites (site 7). *Dermacentor reticulatus* and *D. marginatus* were most frequent on pastures and meadows (sites 5 and 6).

**Figure 2:**
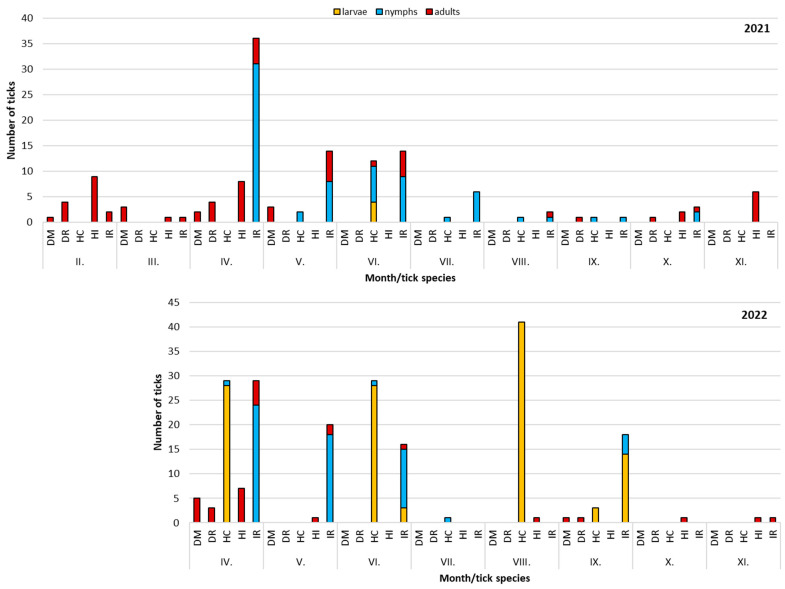
Seasonal dynamics of tick activity at study sites in Žemberovce and Horšianska Dolina National Nature Reserve in 2021–2022. IR—*I. ricinus*, DR—*D. reticulatus*, DM—*D. marginatus*, HC—*H. concinna*, and HI—*H. inermis*. Note: due to low numbers or absence of ticks in some of the sites, data for tick activity are pooled for the whole study area.

Regarding seasonal occurrence, peaks of activity of *H. inermis*, *D. reticulatus* and *D. marginatus* adults were recorded at the end of winter and in early spring, of *I. ricinus* adults and nymphs from April to June. *Haemaphysalis concinna* nymphs occurred from April to September, and adults in June (Figure 2). In both years, there was a short period of overlap of activities of *I. ricinus*, *Dermacentor* spp. and *H. inermis* in April.

#### Tick-Borne Pathogens in Questing Ticks

*Borrelia burgdorferi* s.l. was detected only in *I. ricinus* ticks, in 25.8% (42/163) of the tested samples from sites 3–7 (Table 3, Table 4 and Table 5). Four species, *Borrelia afzelii*, *Borrelia burgdorferi* sensu stricto (s.s.), *Borrelia garinii*, and *Borrelia valaisiana*, were determined with the dominance of *B. afzelii* (47.6%). *Borrelia afzelii* and *B. valaisiana* were present in ticks from all sites, while *B. garinii* was present in sites 4, 6 and 7, and *B. burgdorferi* s.s. was found only in site 7 (Table 5). Genotyping of borreliae was not successful in four nymphs and one female. Nine 5S–23S (rrfA-rrlB) rDNA gene sequence variants of *B. afzelii*, four variants of *B. garinii*, three variants of *B. valaisiana,* and one variant of *B. burgdorferi* s.s. were identified in the tested ticks (for details see Supplementary Table S1).

*Borrelia miyamotoi* was found in eleven *I. ricinus* ticks (6.7%; 11/163) (Table 3 and Table 4) and was present in sites 3, 4, 5, 6 and 7.

*Rickettsia helvetica* and *A. phagocytophilum* were detected in 4.3% (7/163) and 1.2% (2/163) *I. ricinus* ticks, respectively (Table 3 and Table 4). *Anaplasma phagocytophilum* was present in sites 6 and 7, while *R. helvetica* was found in sites 4 and 7.

*Babesia crassa* was detected in *H. inermis* from site 6, *Babesia microti* in *I. ricinus* from site 7 and *Rickettsia raoultii* in *D. marginatus* from site 6; each of the pathogens was found only in a single tick (Table 3 and Table 4). The results represent the first confirmed finding of *B. crassa* in Slovakia.

Co-infections with two pathogens were detected in eight *I. ricinus* ticks: *B. microti* + *B. afzelii* and *R. helvetica* + *B. valaisiana* in one tick each (both in site 7), *B. afzelii + B. miyamotoi* in five ticks (sites 5, 6, 7), and *B. afzelii + B. miyamotoi* from one tick (site 3). No pathogens were found in *H. concinna* and *D. reticulatus* ticks.

Pathogen-positive ticks were found in study sites 3–7. The highest number of positive ticks as well as the highest diversity of pathogens (*A. phagocytophilum*, *B. microti*, four *B. burgdorferi* s.l. species, *B. miyamotoi*, *R. helvetica*) were found at site 7 (Horšianska Dolina National Nature Reserve), while the lowest number of infected ticks and the lowest diversity of pathogens (*B. afzelii*, *B. valaisiana*, and *B. miyamotoi)* were recorded at study sites 3 (at the Sikenica River) and 5 (meadow). In site 4 (vineyard), the presence of five pathogens, three *Borrelia* species (see Table 3 and Table 5), *B. miyamotoi*, and *R. helvetica* was found. The presence of seven pathogen species (three *Borrelia* species, *B. miyamotoi*, *A. phagocytophilum*, *B. crassa*, and *R. raoultii*) was recorded from site 6 (hunter’s cottage) (Table 3).

### 3.2. Tick Infestation in Game Animals

A total of 33 game animals of three species were examined, including 13 red deer, two roe deer, and 18 wild boars. Altogether, 211 feeding ticks were collected, belonging to five species: *D. marginatus*, *D. reticulatus*, *H. concinna*, *H. inermis* and *I. ricinus*. The most common tick species was *I. ricinus* (50.2%) (56 females, 19 males, and 31 nymphs), followed by *D. marginatus* (15 females and 14 males), *D. reticulatus* (22 females and 11 males), *H. concinna* (11 females, 2 males and 8 nymphs), and *H. inermis* (13 females and 9 males). The hunting grounds were located in three distinct biotopes (river, meadow, or field). Ticks were collected from all examined animals except for one wild boar and one roe deer (Table 6).

The mean tick infestation intensity was 7 ± 6.1 ticks per animal in wild boar, 6.0 ± 1.9 ticks per animal in red deer and 3.5 ± 3.3 ticks per animal in roe deer. The highest tick species diversity was recorded on wild boars, with five tick species (*D. marginatus*, *D. reticulatus*, *H. concinna*, *H. inermis*, and *I. ricinus*). Three species were found on red deer (*H. concinna*, *H. inermis*, and *I. ricinus*), whereas only one species was detected on roe deer (*I. ricinus*). *Ixodes ricinus* was most frequently found on red deer. The *Dermacentor* species were predominantly found on wild boar, while *H. inermis* and *H. concinna* showed a preference for cervids (Table 3).

#### Tick-Borne Pathogens in Ticks Feeding on Game Animals

All engorged ticks were tested for the presence of *B. miyamotoi*, *Babesia* spp./*Theileria* spp., *A. phagocytophilum*, *Rickettsia* spp.

The presence of *Borrelia miyamotoi* was detected in two *I. ricinus* ticks from wild boar (Table 7).

*Anaplasma phagocytophilum* was found in 43.1% of feeding ticks (64.2% of *I. ricinus*, 38.1% of *H. concinna*, 18.2% of *H. inermis*, 18.2% of *D. reticulatus*, and 17.2% of *D. marginatus*) removed from all ungulate species (Table 3 and Table 7).

The overall prevalence of *Rickettsia*-positive ticks comprising *I. ricinus*, *H. inermis* and *D. reticulatus* was 4.7% (Table 7). *Rickettsia helvetica* was found in two *I. ricinus* and one *H. inermis* removed from wild boar, and in four *I. ricinus* removed from red deer. *Rickettsia raoultii* was detected in three (9.1%) *D. reticulatus* ticks removed from wild boar (Table 3).

*Babesia*/*Theileria* spp. were found in 16.6% of ticks (Table 7). *Theileria* spp. predominated and were detected in *I. ricinus* (31.5%), *H. inermis* (66.7%), and *H. concinna* (33.3%), all collected from cervids. In DNA isolates from 14 ticks, the sequences were of low quality and were excluded from further analysis. In the remaining ticks, *Theileria capreolii* was identified.

*Babesia microti* was detected in two *I. ricinus*, *Babesia crassa* in one *H. concinna*, an unrecognized species assigned as *Babesia* sp. GNT1 was found in two *H. concinna* and one *D. reticulatus* collected from wild boar. The presence of an unrecognized species assigned as *Babesia* sp. GNT2 was detected in one *H. concinna* removed from red deer (Table 3 and Table 7).

Co-infection of *A. phagocytophilum* and piroplasmids was detected in 10.4% of feeding ticks: with *T. capreoli* in 17 *I. ricinus*, three *H. concinna*, and one *H. inermis*, and with *B. crassa* in one *H. concinna* female.

### 3.3. Tick-Borne Pathogens in Spleens of Game Animals

A total of 33 spleen samples were obtained from game animals (18 from wild boar, 13 from red deer, and 2 from roe deer). Piroplasmids were detected only in the spleen of cervids (Table 3 and Table 8), with an overall prevalence rate of 93.3%. *Theileria capreoli* could be identified in 78.6% samples, while, due to the low quality of the sequences in three DNA isolates, *Theileria* could not be identified to species level (21.4%).

*Anaplasma phagocytophilum* was recorded in 55.6% of wild boar spleens and in 100% of red deer and roe deer spleens (Table 8). Coinfection of piroplasmids with *A. phagocytophilum* was detected in 14 cervids (93.3%).

Eight out of the ten wild boars infected with *A. phagocytophilum* also carried ticks infected with this pathogen. However, the presence of *A. phagocytophilum* was also confirmed in ticks removed from three out of eight negative wild boars.

Thirteen out of the fifteen *A. phagocytophilum*-infected cervids carried ticks infected with *A. phagocytophilum*. Ticks infected with *Theileria* sp. were removed from nine infected cervids. One *A. phagocytophilum*- and *T. capreoli*-infected roe deer did not carry any ticks.

### 3.4. Tick Infestation in Sheep

A total of 48 ticks belonging to three species were collected from 20 sheep. *Dermacentor marginatus* was the dominant species (87.5%), followed by *I. ricinus* (10.4%) and one *H. concinna* (2.1%). The mean tick infestation in sheep was 2.4 ± 2.11 ticks per animal.

*Anaplasma phagocytophilum* was detected in 40% of feeding *I. ricinus* ticks and in 4.8% of *D. marginatus* ticks. *Rickettsia slovaca* was detected in three (7.14%), *R. raoultii* in one (2.4%), and *Anaplasma ovis* in two (4.8%) *D. marginatus* ticks. In the only *H. concinna*, no pathogens were recorded (Table 9).

### 3.5. Principal Component Analysis

**Figure 3:**
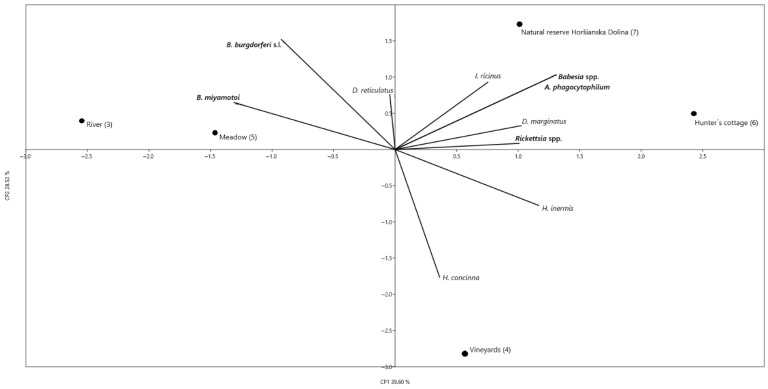
Correlations between questing tick species (*I. ricinus*, *H. concinna*, *H. inermis*, *D. marginatus*, and *D. reticulatus*) specimen numbers, pathogen (*A. phagocytophilum*, *Babesia* spp., *B. burgdorferi* s.l., *B. miyamotoi*, and *Rickettsia* spp.) prevalence in ticks, and study sites (3–7). Principal component analysis (PCA) plot showing the distribution of samples. The first two principal components (PC1 and PC2) explain 68.12% of the total variance.

PCA revealed a high affinity of questing *I. ricinus* for site 7 (natural reserve), whereas *H. concinna* and, to a lesser extent, *H. inermis*, showed preference for site 4 (vineyard) (Figure 3). *Dermacentor marginatus* showed a preference for site 6 (hunter’s cottage), while the preference of *D. reticulatus* for a study site was not significant. As for the correlation of pathogens with sites, it was significant in the case of *B. burgdorferi* s.l. and *B. miyamotoi* in relation to site 3 (river) and site 5 (meadow). This could be explained by the increased movement of reservoir hosts for borreliae. For the other pathogens, correlations were shown with the Nature Reserve Horšianska Dolina (site 7) and the hunter’s cottage (site 6). This also agreed with the occurrence of their vectors, *I. ricinus* for *A. phagocytophilum* and *Babesia* spp., and *D. marginatus* for *Rickettsia* spp.

**Figure 4:**
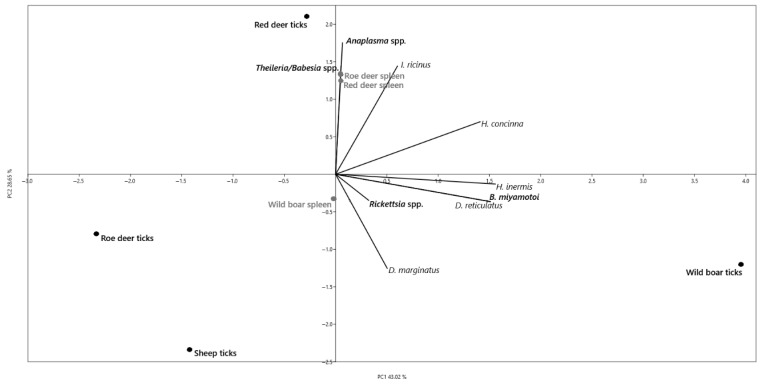
Correlation between the number of feeding tick species (*I. ricinus*, *H. concinna*, *H. inermis*, *D. marginatus*, *D. reticulatus*), number of specimens, and their preferred hosts (red deer, roe deer, wild boar, and sheep); number of tick specimens and pathogen (*Anaplasma* spp., *Theileria*/*Babesia* spp., *B. miyamotoi*, *Rickettsia* spp.) prevalence in feeding ticks and animal tissue (red deer spleen, roe deer spleen, and wild boar spleen). Principal component analysis (PCA) plot showing the distribution of samples. The first two principal components (PC1 and PC2) explain 71.71% of the total variance.

The PCA that involved animals showed a high preference of *I. ricinus* for red deer and, on the contrary, of *D. marginatus* for sheep and wild boar, which are also preferred by *D. reticulatus* (Figure 4), probably due to the environment in which the hosts occur. The presence of *A. phagocytophilum* in cervids correlated to a high degree with the infestation of the animals with *I. ricinus* due to the fact that wild ruminants represent reservoir hosts for *A. phagocytophilum*. The presence of *Theileria*/*Babesia* spp. correlated with the high infection rates in the spleens of cervids. The overall correlation between the presence of *I. ricinus* and *H. concinna*, cervids and the pathogens *A. phagocytophilum* and *Theileria*/*Babesia* spp. confirms the vector–host relationships in this environment, where *I. ricinus* represents the main vector for *A. phagocytophilum*, *H. concinna* is probably the vector of *Theileria* spp., and cervids are the reservoir hosts for these pathogens.

### 3.6. Phylogenetic Analyses

#### 3.6.1. *Anaplasma phagocytophilum*

**Figure 5:**
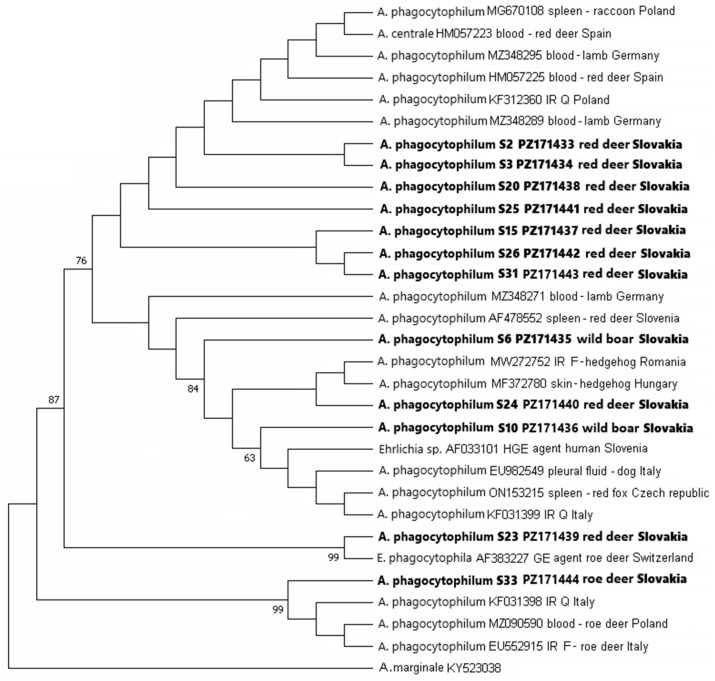
Phylogenetic tree of *groEL* gene sequences of *Anaplasma phagocytophilum* constructed by using the maximum likelihood method based on the Tamura 3-parameter model. The obtained partial DNA sequences (in bold) were compared with sequences from the NCBI database. The bootstrap values above 50% based on 1000 replicates are displayed next to the branches. The partial DNA sequence of *Anaplasma marginale* (NCBI database) was used as an outgroup. IR—*Ixodes ricinus* and Q—questing. Sequences obtained from the spleens of game animals in this study are assigned with an “S” and GenBank accession numbers.

**Figure 6:**
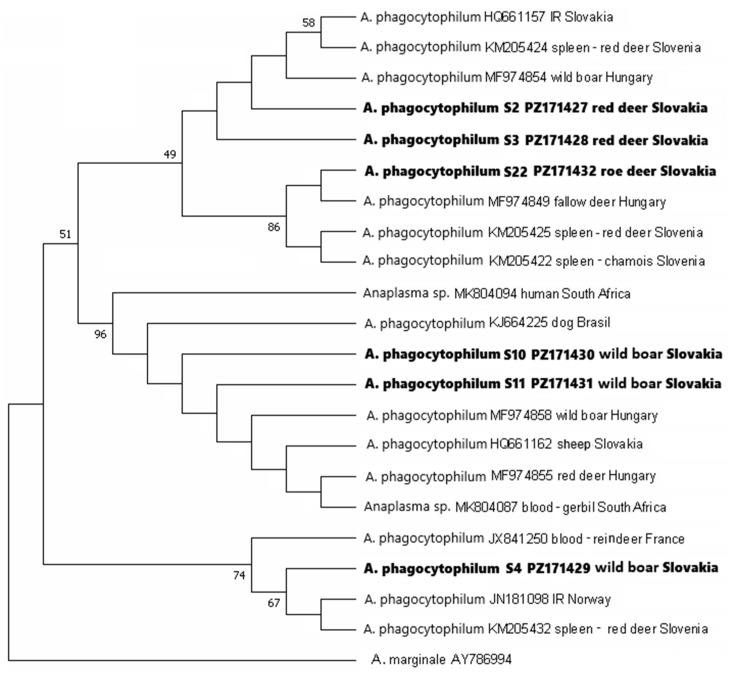
Phylogenetic tree of *msp*4 gene sequences of *Anaplasma phagocytophilum* constructed by using the maximum likelihood method based on the Kimura 2-parameter model + Gamma distribution. The obtained partial DNA sequences (in bold) were compared with sequences from the NCBI database. The bootstrap values above 50% based on 1000 replicates are displayed next to the branches. The partial DNA sequence of *Anaplasma marginale* (NCBI database) was used as an outgroup. IR—*Ixodes ricinus*. Sequences obtained from the spleens of game animals in this study are assigned with an “S” and GenBank accession numbers.

A total of twelve *groEL* sequences (nine from red deer, one from roe deer, and two from wild boar) and six *msp4* sequences (two from red deer, one from roe deer, three from wild boar) of *A. phagocytophilum* were amplified in DNA samples from the spleens of game animals (Figure 5 and Figure 6). The list of sequences is available in Supplementary Table S1.

In the phylogenetic tree constructed based on the 12 *groEL* sequences obtained in this study and 18 partial *groEL* sequences of *A. phagocytophilum* retrieved from GenBank, sequences from red deer and wild boar clustered together with sequences from different sources (wild ruminants, carnivores, sheep, hedgehog, questing *I. ricinus*) (Figure 5). Sequences S2 and S3 from roe deer were identical. The percentage of identity of the *groEL* sequences from red deer and wild boar with reference sequences from the GenBank was 99–100%. The sequence S10 from wild boar was identical to the isolate from the blood of an HGA patient from Slovenia (AF033101). The sequence S33 from roe deer formed a separate cluster with sequences from roe deer, questing ticks, and ticks feeding on roe deer. It was 100% identical to the Italian isolate from a questing *I. ricinus* (KF031398), a Polish isolate from roe deer blood (MZ090590), and an Italian isolate from *I. ricinus* fed on roe deer (EU552915). The sequence was 99.62% identical to sequence KF383237 from a questing *I. ricinus* from eastern Slovakia.

In the phylogenetic tree constructed based on the six *msp*4 sequences from this study and 15 partial *msp*4 sequences of *A. phagocytophilum* retrieved from GenBank, sequences S2 and S3 from red deer and S4, S10, S11 from wild boar clustered together with sequences from tissues of wild ruminants, wild boar, sheep, dog, and ticks, including an *I. ricinus* (HQ661157) and sheep blood (HQ661162) from Slovakia (Figure 6). The percentage of identity of the *msp*4 sequences from red deer and wild boar with reference sequences from the GenBank was 99–100%. Sequences S10 and S11 obtained from a wild boar were identical with each other and also with the Slovak isolate from sheep (HQ661162). The sequence S4 from wild boar was identical with an isolate from *I. ricinus* from Norway (JN181098) and an isolate from red deer spleen from Slovenia (KM205432). Sequence S22 from roe deer spleen was identical to the Slovenian isolate from red deer spleen (KM205425) and chamois spleen (KM205422).

#### 3.6.2. *Anaplasma ovis*

**Figure 7:**
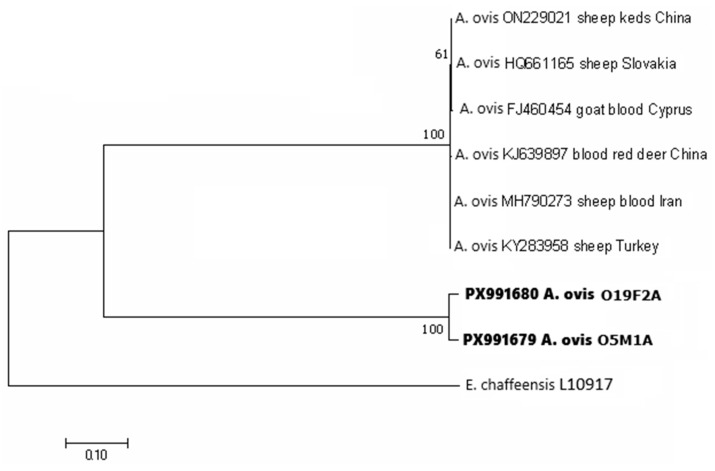
Phylogenetic tree of *msp*4 gene sequences of *Anaplasma ovis* constructed by using the maximum likelihood method based on the Tamura 3-parameter model. The obtained partial DNA sequences (in bold) were compared with sequences from the NCBI database. The bootstrap values based on 1000 replicates are displayed next to the branches. The partial DNA sequence of *Ehrlichia chaffeensis* (NCBI database) was used as an outgroup. O19F2 and O5MI sequences were obtained in this study from *Dermacentor marginatus* females and males, respectively, feeding on sheep.

Two *msp4* sequence variants of *A. ovis* were identified in adult *D. marginatus* ticks removed from sheep. In the phylogenetic tree constructed based on the two *msp4* sequences from *D. marginatus* and six partial *msp4* sequences of *A. ovis* retrieved from GenBank, two well-supported clusters were formed. The sequences from *D. marginatus* were 99.39% identical and clustered together, while the reference sequences from sheep, goat, red deer and sheep ked formed a separate cluster (Figure 7). The sequences O5M1A and O19F2A were 98.60% and 99.29% identical to the Slovak isolate HQ661165 from sheep, respectively, and 98.29–99.29% identical with the other reference sequences.

#### 3.6.3. *Rickettsia* Species

**Figure 8:**
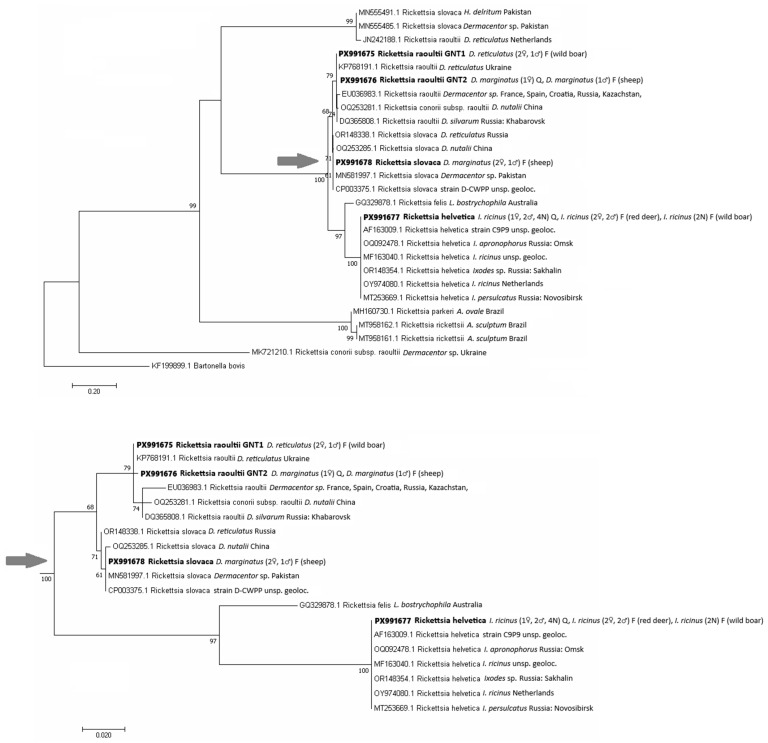
Phylogenetic tree of *sca*4 gene sequences of *Rickettsia* species constructed by using the maximum likelihood method based on the Tamura 3-parameter model with invariant sites. The obtained partial DNA sequences (in bold) were compared with sequences from the NCBI database. The bootstrap values based on 1000 replicates are displayed next to the branches. The partial DNA sequence of *Bartonella bovis* (NCBI database) was used as an outgroup. Q—questing, F—feeding, and N—nymph. In parentheses next to the identified genotypes, the host of the feeding ticks, the developmental stage and the number of the analyzed ticks are given. The arrow indicates the node where the subtree shown below begins.

One *sca*4 gene sequence variant of *R. helvetica* was identified in 13 *I. ricinus* ticks (questing and feeding on red deer and wild boar), and one sequence variant of *R. slovaca* was found in two *D. marginatus* ticks removed from sheep. Two sequence variants of *R. raoultii* were recorded: GNT1 variant in three *D. reticulatus* removed from wild boar and GNT2 variant in *D. marginatus* retrieved from vegetation and sheep (Figure 8). The list of sequences is available in Supplementary Table S1.

The phylogenetic tree was constructed based on four *sca*4 sequences from this study and 22 *sca*4 sequences of *Rickettsia* species retrieved from GenBank. The sequence of *R. helvetica* clustered together with other *R. helvetica* sequences derived from *Ixodes* spp. ticks from Europe and Russia, and was 100% identical with the sequence MF163040 of the AS819 isolate from Germany. The sequence of *R. slovaca* clustered together with sequences obtained from *Dermacentor* spp. ticks from Russia, China or Pakistan, and was 100% identical with part of the complete genome of *R. slovaca* CP002428.

The two identified genotypes of *R. raoultii* were 99.83% identical and clustered together with *R. raoultii* sequences derived from *Dermacentor* spp. from different European countries: Russia and China. Variant GNT1 was 100% identical with the sequence KP768191 isolated from questing *D. reticulatus* from Ukraine, while variant GNT2 was 100% identical with the sequence MT253668 isolated from *D. marginatus* from Russia.

#### 3.6.4. *Babesia* and *Theileria* Species

Analysis of the genetic variability of the 18S rRNA gene sequences revealed the presence of one variant of *B. microti* in *I. ricinus* (one questing and one removed from wild boar) and of one variant of *B. crassa* in one questing *H. inermis* and one *H. concinna* feeding on wild boar. A sequence variant of a yet unidentified species, assigned in this study as *Babesia* sp. GNT1 was found in two *H. concinna* and one *D. reticulatus* feeding on wild boar. Another variant of an unidentified species, *Babesia* sp. GNT2, was identified in a *H. concinna* removed from red deer. The presence of two sequence variants of *T. capreoli* was confirmed: *T. capreoli* GNT1 identified in a red deer spleen and in two *I. ricinus* removed from red deer, and *T. capreoli* GNT2 was identified in the spleens of three red deer and of two roe deer. The list of sequences is available in Supplementary Table S1 and sequences in fasta format at https://doi.org/10.6084/m9.figshare.31869478.

**Figure 9:**
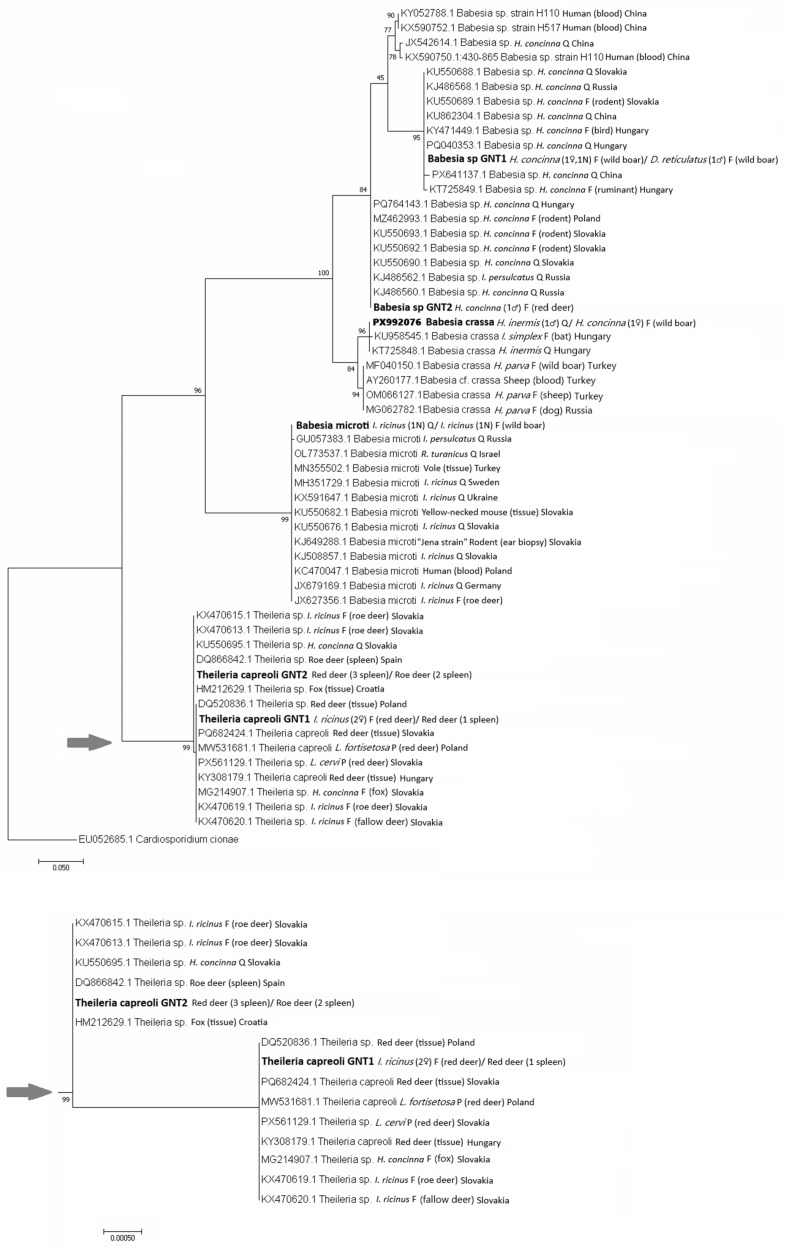
Phylogenetic tree of 18S rRNA gene sequences of *Babesia* and *Theileria* species constructed by using the maximum likelihood method based on the Tamura 3-parameter model with Gamma distribution. The obtained partial DNA sequences (in bold) were compared with sequences from the NCBI database. The bootstrap values based on 1000 replicates are displayed next to the branches. The partial DNA sequence of *Cardiosporidium cionae* (NCBI database) was used as an outgroup. Q—questing, F—feeding, P—parasitizing, and N—nymph. In parentheses next to the identified genotypes, the host of the feeding ticks, the developmental stage, and the number of the analyzed ticks are given. The arrow indicates the node where the subtree shown below begins.

The phylogenetic tree (Figure 9) was inferred from the six *Babesia* 18S rRNA gene sequence variants identified in this study, 37 18S rRNA sequences of *Babesia* species, and 13 *Theileria* species retrieved from GenBank. The analysis showed a 100% identity of the *Babesia* sequence isolated from *Haemaphysalis* spp. with *B. crassa* from *H. inermis* (KT725848) from Hungary, and an identity of 98.63–99.76% with *B. crassa* isolates from *Haemaphysalis* species from Turkey and Russia, and from sheep blood from Turkey. The sequence of *Babesia* sp. GNT1 segregated with a highly significant bootstrap support with other unidentified *Babesia* species known from *H. concinna* from Slovakia, Hungary, Russia and China (assigned as *Haemaphysalis* sp. hc hlj212). The sequence of *Babesia* sp. GNT2 was placed in a cluster with unrecognized *Babesia* species from *H. concinna* from Slovakia, Hungary, Poland, and Russia, as well as from an *I. persulcatus* from Russia (*Babesia* sp. Kh-Hc232) (Figure 9). The *B. microti* sequence was placed with a highly significant bootstrap support into the *B. microti* clade. It was identical with isolates from *I. ricinus* ticks and rodents from Slovakia. Importantly, the obtained sequence was 100% identical to the *B. microti* EF413181 strain Jena isolated from human blood in Germany. The *T. capreoli* sequence variants GNT1 and GNT2 from this study segregated in the *Theileria* clade jointly with sequences of *T. capreoli* isolates from cervids from Slovakia, Poland and Hungary, from *I. ricinus* feeding on deer in Slovakia, or from deer ked from Slovakia and Poland (Figure 9). The sequences are identical, with the previously identified sequences of *Theileria* sp. 1 and *Theileria* sp. 2 from Slovakia.

## 4. Discussion

We explored an area in south-western Slovakia, where the distribution ranges of five epidemiologically important tick species overlap. Such sites are rare in Slovakia as well as in other regions of Central Europe and give us the opportunity to explore more complex tick–pathogen–vertebrate host relationships than in sites where single or two tick species co-occur. Considering this fact, we expected the occurrence of a broad spectrum of tick-borne pathogens associated with the tick species found in Žemberovce and environs. In a previous research in this study area, two nidicolous *Ixodes (Pholeoixodes)* species, *I. kaiseri* and *I. canisuga*, associated with red fox and European badger, were found [45].

In Slovakia, 25 tick species have been recorded till present [46]. Among them, *I. ricinus* and *D. reticulatus* are the most widespread and most significant from a medical and veterinary point of view. However, *D. marginatus*, *H. concinna* and *H. inermis* were also found to be involved in the circulation of zoonotic pathogens [4]; thus, their presence in an area should not be neglected.

Although there is a general trend towards the spread of ticks into green urban areas in Europe, where abundant populations of *I. ricinus* are present [47], two of the study sites located in the residential zone of Žemberovce were free of ticks, probably due to unfavorable microclimatic conditions, or due to the absence of suitable reservoir hosts (e.g., small mammals). In contrast, in green areas (as the semi-natural refugees) of other cities in Slovakia, presence of *I. ricinus* populations has been confirmed, e.g., in Bratislava [20,48,49], Malacky [50], Bardejov and Košice [36]. In Košice, *I. ricinus* was found to co-occur with *D. reticulatus* and *D. marginatus* [23]. The presence of ticks in the other study sites in Žemberovce is in accordance with the known requirements of the individual species to the type of habitat and microclimatic conditions [4,51]. *Ixodes ricinus* was most abundant in the natural deciduous forest of Horšianska Dolina Nature Reserve, while *Dermacentor* spp. preferred sites in the rural area (pastures, meadows). This preference of individual tick species to certain habitat types was also confirmed by PCA (see Figure 3). According to the literature, *Haemaphysalis* spp. can be found in different habitats, e.g., deciduous forests, forest clearings, river banks, ecotones [4,51]. In this study, they showed a preference for site 4 (vineyard), but were also found in the rural sites 5 and 6 (meadow and hunter’s cottage). With regard to the seasonal activity of the individual species, they were consistent with previous findings—*Dermacentor* spp. and *H. inermis* were active in colder months, *H. concinna* occurred during summer. The prolonged activity of *I. ricinus*, also covering late autumn and February, was also confirmed and is consistent with findings on the winter activity of the species [52]. From an epidemiological point of view, both spatial and temporal overlap of the occurrence of sympatric tick species are important. In Žemberovce, all five species were present in site 6 (hunter’s cottage), but at the Sikenica river bank, only *I. ricinus* and *D. reticulatus* were present. However, the spread of the recorded tick species by birds and larger mammals within the whole rural zone of Žemberovce is still possible. With regard to the temporal overlap of activities of the tick species, April was the month when four tick species were active (*I. ricinus*, *H. inermis*, and both *Dermacentor* spp.). Considering the fact that these ticks are known to share the same hosts, which was also confirmed in this study, co-feeding transmission and spillover of pathogens between multiple tick species may occur.

As expected, a considerable diversity of tick-borne pathogens was revealed in the study area. In total, the occurrence of 15 pathogens associated with the vector ticks and reservoir hosts present in the study sites was confirmed. Out of them, ten species were detected in questing ticks (four *B. burgdorferi* s.l. species, *B. miyamotoi*, *R. helvetica*, *R. raoultii*, *A. phagocytophilum*, *B. crassa*, and *B. microti*), seven species (*B. miyamotoi*, *R. helvetica*, *R. raoultii*, *A. phagocytophilum*, *B. crassa*, *B. microti*, and *T. capreoli*) and genotypes of two yet-to-be-described *Babesia* spp. were found in ticks removed from wild ungulates, two types of pathogens (*A. phagocytophilum* and *Theileria* spp.) were detected in the spleen of wild ungulates, and four pathogens (*A. phagocytophilum*, *A. ovis*, *R. raoultii*, and *R. slovaca*) in ticks that were removed from sheep. We assume that the number of pathogens circulating in Žemberovce may be even higher, as some pathogenic species associated with ticks (e.g., Tick-borne encephalitis virus, *Francisella tularensis*, and *Coxiella burnetii*) were not included in the panel of screened microorganisms. Though we admit the following limitation, we did not screen ticks removed from cervids and wild boar for *B. burgdorferi* s.l., since wild ungulates are not considered reservoir hosts for *B. burgdorferi* s.l. [53], and only a few ticks, positive for this group of spirochaetes, were found on ungulates in a previous study [54]. Cervids have been suggested as dilution hosts [49]; however, they can serve as sentinels to monitor the presence of ticks and tick-borne pathogens in the environment [55].

Four *Rickettsia* species (*R. helvetica*, *R. monacensis*, *R. slovaca*, and *R. raoultii*) [56], three *B. burgdorferi* s.l. species, *A. phagocytophilum*, *B. microti* and *B. venatorum* [27], and the spillover of some species to ticks that are not considered their vectors, e.g., *R. raoultii* to *H. concinna* and *H. inermis* [57], as well as *B. burgdorferi* s.l. to *D. reticulatus* and *H. inermis* [27], were reported from the Slovak Karst (eastern Slovakia), where the same exophilic species as in this study, plus the nidicolous *Ixodes trianguliceps*, were found.

The same *Rickettsia* species as in this study (*R. helvetica*, *R. slovaca*, *R. raoultii,* and, in addition, *R. monacensis*) were identified in a site in south-western Slovakia with sympatric occurrence of *I. ricinus*, *D. reticulatus*, and *H. concinna* [17]. In the Bratislava green areas and the deciduous forests of the Small Carpathian Mountains, where *I. ricinus* co-occurs with *H. concinna*, a wide spectrum of pathogens was found in questing ticks as well as ticks feeding on wild ungulates [21,54,58,59,60,61]. These comprised six *B. burgdorferi* s.l. species, *B. miyamotoi*, *A. phagocytophilum*, *R. helvetica*, *R. monacensis*, *B. microti*, *B. venatorum*, *B. capreoli*, *B.* cf. *odocoilei*, two undescribed *Babesia* spp. from *H. concinna*, *Neoehrlichia mikurensis*, a *B. motasi*-like species, *T. capreoli,* and *C. burnetii*.

Unusual tick–pathogen association indicating spillover either by co-feeding or from infected hosts was also recorded in this study, particularly in the case of *A. phagocytophilum* detected in *Haemaphysalis* spp. and *D. reticulatus*, and *R. helvetica* in *H. concinna* removed from wild ungulates, as well as in the case of *A. phagocytophilum* in *D. marginatus* removed from sheep.

### 4.1. Borrelia Species

The overall mean prevalence of *Borrelia burgdorferi* s.l. in *I. ricinus* ticks in Europe is 13.7%, according to a meta-analysis by Rauter and Hartung [62] and 18.8% in Slovakia [63]. A higher infection prevalence (25.8%) was recorded in *I. ricinus* ticks collected in Žemberovce; however, 163 questing ticks were tested.

The distribution of *B. burgdorferi* s.l. species depends on the presence of suitable reservoir hosts. In total, four species were identified in this study. *Borrelia afzelii*, *B. garinii* and *B. valaisiana* are among the most frequently reported species in Slovakia [63] and Central Europe [64]. *Borrelia burgdorferi* s.s. is less frequent, consistent with findings from previous studies [4,61]. In this study, this species was confirmed only in one tick. Three of the recorded species, except *B. valaisiana*, are known to be pathogenic to humans [65,66] and may represent a risk for inhabitants of Žemberovce and hunters and forest workers in the region. High interspecific variability was revealed by analyzing the 5S–23S (rrfA-rrlB) rRNA intergenic spacer. As the aim of this study was primarily the identification of *B. burgdorferi* s.l. to species level, the selected approach was suitable. We are aware of the fact that for deeper interspecific phylogenetic analyses of species within the *B. burgdorferi* s.l. complex, MLST-based typing is more appropriate, as it involves sequencing of eight shared housekeeping genes (clpA, clpX, nifS, pepX, pyrG, recG, rplB, and uvrA) [67]. However, such a comprehensive approach was beyond the scope of this study.

The 6.8% prevalence of *B. miyamotoi* in questing *I. ricinus* determined in this study was higher than in previous studies from Slovakia [50,68], which may be connected with the presence and abundance of small rodents serving as reservoir hosts. However, we have no data on the populations of small rodents from the study area. Nevertheless, people in the studied region may be at risk of infections with this pathogen.

### 4.2. Anaplasma Species

The prevalence of *A. phagocytophilum* in questing *I. ricinus* from the study area was surprisingly low (1.2%) in comparison with data from other sites in Slovakia [48,58]. The results are even more surprising for an area where reservoir hosts like cervids or wild boar occur. Moreover, infection rates in game animals (up to 100%) and in ticks feeding on them (43%) were high in the study area, and we assume that infected game also range in the natural reserve and in the rural zone of Žemberovce. The high prevalence of game infected with the bacterium in the study area is in agreement with previous findings from south-western Slovakia [54].

Several genotypes of *A. phagocytophilum* were identified in cervids and wild boar in the study area based on the analysis of the *groEL* and *msp*4 genes. In general, the genetic variability of European strains of *A. phagocytophilum* is very high [69,70,71], and the circulation of a number of *A. phagocytophilum* strains was also confirmed in different sites of Slovakia [54,72,73,74,75]. In the phylogenetic analysis, genotypes from the present study were placed in different subclusters of the phylogenetic trees for the *groEL* and *msp*4 sequences and clustered together with sequences of isolates from the blood of domestic animals, deer and wild boar, but also with isolates from human blood. Based on these analyses, we assume that some of the genotypes that circulate in the area of Žemberovce might represent a risk for livestock, and some are non-pathogenic and associated with roe deer. However, the current results do not allow us to conclude whether some of the genotypes may represent a risk for humans. The results of the study point to the necessity to perform more robust genetic analyses to elucidate the complex associations of *A. phagocytophilum* genotypes circulating in the study area with reservoir hosts and their pathogenicity to wildlife, livestock and humans.

Anaplasmosis is the most common tick-borne bacterial disease of livestock and free-living ungulates in Europe [76]. *Anaplasma ovis* represents a microorganism that is infective for sheep and goats [39]. *Dermacentor marginatus* is the main vector of this bacterium in Central Europe [72,77]. In our study, 4.8% (2/42) of the tested *D. marginatus* ticks were positive for *A. ovis*. The two obtained sequences in our study were identical to a Slovak isolate previously obtained from sheep blood samples [72]. In another study from Slovakia, *A. ovis* was detected in 8.2% of tested sheep flocks [73]. Our findings suggest the presence of an endemic focus for ovine anaplasmosis in the study area and indicate the need for further studies analyzing also the animals and a larger number of ticks.

### 4.3. Rickettsia Species

Rickettsial diseases are generally zoonotic, affecting both humans and animals, with mammals sometimes acting as reservoir hosts for these pathogens [78]. In particular, wild ungulates play a key role as hosts for both the immature and the adult ticks, and may facilitate the maintenance and circulation of rickettsiae within enzootic cycles.

*Rickettsia*-specific DNA has been detected in questing and feeding ticks, and in the blood and tissues of various vertebrate hosts, including roe deer and wild boar. In addition, antibodies against rickettsiae have been reported (among other animals) in red deer, roe deer and wild boars [55], which were studied in the recent work. Ticks (*I. ricinus*, *D. reticulatus*, and *D. marginatus*) are vectors and reservoirs of rickettsiae of the spotted fever group (SFG) in Slovakia [78], where six SFG rickettsial species transmitted by ticks have been identified: *Rickettsia helvetica*, *R. slovaca*, *R. raoultii*, *R. monacensis*, *R. conorii* and *R. africae* [78,79,80]. Three of these rickettsiae were determined in our study. *Ixodes ricinus* is the main reservoir for *R. helvetica* [81]. In questing ticks from Žemberovce, only *R. helvetica* was detected in *I. ricinus* ticks (4.3%), while this species was also detected in single *H. inermis* ticks feeding on red deer and wild boar, respectively. *Rickettsia helvetica* is the most commonly found *Rickettsia* species in Slovakia [56,60] and is considered pathogenic due to multiple reports of clinical symptoms like fever, headache, and myalgia in connection with *R. helvetica* infections in humans [82,83]. *Dermacentor marginatus* is the main vector of *R. slovaca* in Slovakia [84], which was confirmed in three ticks feeding on sheep in our study. According to previous studies, this tick species is regarded as a potential vector of various bacterial pathogens, including *R. helvetica* and *R. raoultii* [85]. *Rickettsia raoultii* is transmitted mainly by *D. reticulatus* [56], so it is not surprising that we detected an infection in a single tick collected from a wild boar. However, this pathogen was also detected in *D. marginatus* collected from vegetation and from sheep. Our results highlight the involvement of wild ungulates, domestic animals, and, of course, ticks in rickettsiae circulation in the study area, emphasizing the importance of monitoring these interactions to better assess the risk to human health.

### 4.4. Babesia/Theileria Species

The overall prevalence of *Babesia* spp. in questing ticks from Žemberovce was low, but consistent with findings from other sites in Slovakia [21,86]. Two recognized species, *B. microti* and *B. crassa*, and two undescribed species were detected in questing, as well as in engorged ticks. *Babesia microti* was genetically identical with the human pathogenic strain Jena and with previously analyzed Slovak isolates from *I. ricinus* and rodent tissues [21]. The presence of *B. crassa* was confirmed in Slovakia for the first time. *Babesia crassa* was originally described as a sheep parasite from Iran [87]. Subsequently, *B. crassa*-like parasites were detected in sheep, as well as *Ixodes persulcatus* and *H. concinna* ticks in China, along with infections in humans [88]. Recently, human infections caused by the *B. crassa*-like parasites were also confirmed in Slovenia [89] and France [90]. The isolate is genetically identical with the Hungarian isolate from *H. inermis* [91], but its pathogenicity to humans and its epidemiological relevance are not clear, as it is only 95.69% identical with a *B. crassa*-like isolate from human blood in Slovenia [89]. Based on our results, it is not possible to confirm if this parasite is established in Slovakia. There is also a probability that the infected ticks were transported to the study area from Hungary by birds.

The two unrecognized species, *Babesia* GNT1 and *Babesia* GNT2, are identical with the Chinese isolate from *H. concinna* (*Haemaphysalis* sp. hc hlj212) and the isolate from *I. persulcatus* from Russia (*Babesia* sp. Kh-Hc232), respectively. The same species have already been found in south-western Slovakia (*Babesia* sp. 1 (Eurasia) and *Babesia* sp. 2 (Eurasia), respectively) [21] and have also been identified in questing and bird-feeding *Haemaphysalis* spp. in Hungary [92,93]. Findings from Hungary, based on the analysis of a relatively large number of questing *H. concinna* of different developmental stages, suggest that the species may be indigenous in the country [92]. However, it is still questionable if these species are established in *Haemaphysalis* spp. populations in south-western Slovakia, or if they are regularly transported there by birds from Hungary. Regular samplings of questing *Haemaphysalis* spp. ticks and their molecular screening for the presence of piroplasmids should be carried out in Slovakia to shed light on their origin. Interestingly, no *Babesia canis*-infected *Dermacentor* ticks were found, which suggests that the pathogen is not endemic in Žemberovce. However, analyzing a larger sample of ticks and consultations with local veterinarians is needed to confirm this assumption.

Over 50% of examined cervids in Slovakia were found to be infected with *Theileria* sp. [54,94]. Two genotypes of *T. capreoli* have been identified in cervids in the forests of the Small Carpathian Mountains, one associated with roe deer and the other one with red deer and fallow deer [54]. High infection rates with *T. capreoli* were also detected in the examined cervids and attached ticks from Žemberovce. Molecular analyses confirmed the presence of the two previously identified genotypes. In contrast, no *Theileria*-infected questing ticks were found, which is in agreement with previous findings on their very low infection rate [21]. *Haemaphysalis concinna*, which frequently infests cervids, is suggested to be the vector of *T. capreoli* [92], but based on the low numbers of questing ticks that could be screened for *Theileria*, it was not possible to confirm the vector role of *H. concinna* in Slovakia. *Theileria capreoli* is not considered to be zoonotic, but chronic asymptomatic infections have been detected in cervid populations throughout Europe [95,96].

## 5. Conclusions

The results of our research carried out in an area with sympatric occurrence of five epidemiologically important tick species revealed the presence of a high diversity of tick-borne microorganisms. These include four species of the *B. burgdorferi* s.l. complex, *B. miyamotoi*, three *Rickettsia* species, *A. phagocytophilum*, *A. ovis*, four *Babesia* species and *Theileria capreoli.* The presence of *B. crassa*, which was confirmed in Slovakia for the first time, together with the findings of “far-eastern” *Babesia* species in *H. concinna* ticks call for further studies on the origin of these piroplasms and their pathogenicity to humans and animals. Three *B. burgdorferi* s.l. species, *B. miyamotoi*, all three *Rickettsia* species and *B. microti* are zoonotic, while *A. ovis* causes disease in small ruminants and was found only in ticks feeding on sheep. Among the detected pathogens, the highest risk to human health in the region is represented by members of the *Borrelia burgdorferi s.l.* complex, specifically *B. afzelii*, *B. garinii*, and *B. burgdorferi* s.s., as these species are well-established causative agents of Lyme borreliosis in Europe. Given that Lyme borreliosis is the most common tick-borne disease in Europe, the presence of these pathogenic *Borrelia* species suggests a significant public health concern in the region. Consequently, individuals exposed to tick habitats may be at risk of infection, particularly during peak *I. ricinus* tick activity seasons.

A high genetic variability of *A. phagocytophilum* was observed. Some strains are probably pathogenic to livestock, but the results of the phylogenetic analyses did not enable specifying strains pathogenic to humans and further genetic analyses are needed. Infestation of sheep with ticks infected with *A. phagocytophilum* and *A. ovis* suggests that small ruminants in the Žemberovce area may be at risk of infections with anaplasmosis, and therefore regular screening of the animals for the infections is needed.

Reservoir competence of wild cervids was confirmed for *T. capreoli* and *A. phagocytophilum,* and of wild boar for *A. phagocytophilum.* In addition, game animals are probably also involved in the transport and dispersal of ticks infected with zoonotic tick-borne pathogens. This research points to the risk of exposure of residents of the village Žemberovce and of hunters and tourists, as well as of free-grazing livestock, to multiple tick species carrying multiple zoonotic agents. Further studies are necessary to fully understand the complex tick–pathogen–host associations in the study area with implications for public health.

## Figures and Tables

**Table 1:** Sequences of primers and probes used in PCRs for the detection of tick-borne pathogens.

Pathogen	Primer	Oligonucleotide Sequence (5′-3′)	Reference
	Probe		
*Anaplasma phagocytophilum*	ApMSP2f	ATGGAAGGTAGTGTTGGTTATGGTATT	[35]
	ApMSP2r	TTGGTCTTGAAGCGCTCGTA	
	ApMSP2p-FAM probe	TGGTGCCAGGGTTGAGCTTGAGATTG	
	MSP4Ap5f	ATGAATTACAGAGAATTGCTTAGTAGG	[36]
	MSP4Ap3r	TTAATTGAAAGCAAATCTTGCTCCTATG	
	msp4f	CTATTGGYGGNGCYAGAGT	
	msp4r	GTTCATCGAAAATTCCGTGGT	
	EphplgroEL-F	ATGGTATGCAGTTTGATCGC	[37]
	EphplgroEL-R	TTGAGTACAGCAACACCACCGGAA	
*Anaplasma ovis*	msp45	GGGAGCTCCTATGAATTACAGAGAATTGTTTAC	[41]
	msp43	CCGGATCCTTAGCTGAACAGGAATCTTGC	
*Borrelia miyamotoi*	Bmp41f	TTGCTTGTGCAATCATAGCC	[40]
	Bmp41r	GCAAATCTTGGTGCTTTTCAA	
	Bmp41 dye-labeled probe	Cy5AGATGCCACAATTCATCTGTCATTA-BBQ-650	
*Borrelia burgdorferi* s.l.	Bb23Sf	CGAGTCTTAAAAGGGCGATTTAGT	[35]
	Bb23Sr	GCTTCAGCCTGGCCATAAATAG	
	Bb23Sp-FAM probe	AGATGTGGTAGACCCGAAGCCGAGTG	
	IGSa	CGACCTTCTTCGCCTTAAAGC	[39]
	IGSb	AGCTCTTATTCGCTGATGTA	
*Babesia*/*Theileria* spp.	BJ1	GTCTTGTAATTGGAATGATGG	[34]
	BN2	TAGTTTATGGTTAGGACTACG	
*Rickettsia* spp.	D767f	CGATGGTAGCATTAAAAGCT	[38]
	D1390r	CTTGCTTTTCAGCAATATCAC	

**Table 2:** Tick species and numbers collected at five study sites (2021–2022) in Žemberovce and Horšianska Dolina National Nature Reserve. Developmental stages: L—larva, N—nymph, A—adult. For site identification, see Figure 1.

Tick Species	Stage	Collection Sites					Total
		Site 3	Site 4	Site 5	Site 6	Site 7	
*I. ricinus*	L	0	1	0	0	16	17
	N	3	19	16	20	58	116
	A	3	3	6	6	12	30
	Total	6	23	22	26	86	163
*H. concinna*	L	0	73	3	0	0	76
	N	0	3	3	7	4	17
	A	0	0	0	1	0	1
	Total	0	76	6	8	4	94
*H. inermis*	L	0	1	0	0	0	1
	A	0	12	3	21	0	36
	Total	0	13	3	21	0	37
*D. reticulatus*	A	1	1	6	3	3	14
*D. marginatus*	A	1	0	0	14	0	15

**Table 3:** Overview of questing tick species and species infesting game and sheep, presence of tick-borne pathogens in questing and engorged ticks per site and animal host, and in the spleen of game in Žemberovce and Horšianska Dolina National Nature Reserve in 2021–2022. **+** presence of a pathogen. BA—*Borrelia afzelii*, BG—*Borrelia garinii*, BV—*Borrelia valaisiana*, Bss—*Borrelia burgdorferi* s.s., BM—*Borrelia miyamotoi*, AP—*Anaplasma phagocytophilum*, AO—*Anaplasma ovis*, RH—*Rickettsia helvetica*, RR—*Rickettsia raoultii*, RS—*Rickettsia slovaca*, BM—*Babesia microti*, BC—*Babesia crassa*, BG1—*Babesia* sp. GNT1, BG2—*Babesia* sp. GNT2, and TH—*Theileria* including *T. capreoli.* Note: engorged ticks were not tested for *B. burgdorferi* s.l.

	BA	BG	BV	Bss	BM	AP	AO	RH	RR	RS	BM	BC	BG1	BG2	TH
Questing ticks															
Site 3															
*I. ricinus*	**+**		**+**		**+**										
*D. reticulatus*															
*D. marginatus*															
Site 4															
*I. ricinus*	**+**	**+**	**+**		**+**			**+**							
*H. concinna*															
*H. inermis*															
*D. reticulatus*															
Site 5															
*I. ricinus*	**+**		**+**		**+**										
*H. concinna*															
*H. inermis*															
*D. reticulatus*															
Site 6															
*I. ricinus*	**+**	**+**	**+**		**+**	**+**									
*H. concinna*															
*H. inermis*												**+**			
*D. reticulatus*															
*D. marginatus*									**+**						
Site 7															
*I. ricinus*	**+**	**+**	**+**	**+**	**+**	**+**		**+**			**+**				
*H. concinna*															
*D. reticulatus*															
Spleen of game															
Red deer						**+**									**+**
Roe deer						**+**									**+**
Wild boar						**+**									
Feeding ticks															
Red deer															
*I. ricinus*	-	-	-	-		**+**		**+**							**+**
*H. concinna*	-	-	-	-		**+**								**+**	**+**
*H. inermis*	-	-	-	-		**+**									**+**
Roe deer															
*I. ricinus*	-	-	-	-		**+**									
Wild boar															
*I. ricinus*	-	-	-	-	**+**	**+**		**+**			**+**				**+**
*H. concinna*	-	-	-	-		**+**						**+**	**+**		
*H. inermis*	-	-	-	-		**+**		**+**							**+**
*D. reticulatus*	-	-	-	-		**+**			**+**						**+**
*D. marginatus*	-	-	-	-		**+**									
Sheep															
*I. ricinus*	-	-	-	-		**+**									
*H. concinna*	-	-	-	-											
*D. marginatus*	-	-	-	-		**+**	**+**		**+**	**+**					

**Table 4:** Prevalence of pathogens (%) with 95% confidence intervals in questing ticks from five study sites (2021–2022) in Žemberovce and Horšianska Dolina National Nature Reserve. L—larvae, N—nymph, A—adult, and *n*—number. ^a^ *Babesia microti*, ^b^ *Babesia crassa*, ^c^ *Rickettsia helvetica*, and ^d^ *Rickettsia raoultii*.

Tick Species	Stage	Total	*A. phagocytophilum*		*B. burgdorferi* s.l.		*Babesia* spp.		*Rickettsia* spp.		*B. miyamotoi*	
		Tested	Positive *n* (%)	95% CI	Positive *n* (%)	95% CI	Positive *n* (%)	95% CI	Positive *n* (%)	95% CI	Positive *(n*)	95% CI
*I. ricinus*	L	17	0 (0)	0	0 (0)	0	0 (0)	0	0 (0)	0	0 (0)	0
	N	116	1 (0.9)	0.2–4.7	30 (25.9)	18.8–34.5	1 (0.9) ^a^	0.2–4.7	4 (3.4) ^c^	1.3–8.5	8 (7)	3.5–13.0
	A	30	1 (3.3)	0.6–16.7	12 (43.3)	27.4–60.8	0 (0)	0	3 (2.6) ^c^	0.9–7.3	3 (10)	3.5–25.6
	Total	163	2 (1.2)	0.3–4.4	42 (25.8)	19.7–33.0	1 (0.6)	0.1–3.4	7 (4.3)	2.1–8.6	11 (6.8)	3.8–11.7
*H. inermis*	L	1	0 (0)	0	0 (0)	0	0 (0)	0	0 (0)	0	0 (0)	0
	A	36	0 (0)	0	0 (0)	0	1 (2.8) ^b^	0.5–14.2	0 (0)	0	0 (0)	0
	Total	37	0 (0)	0	0 (0)	0	1 (2.8)	0.5–14.2	0 (0)	0	0(0)	0
*D. marginatus*	A	15	0 (0)	0	0 (0)	0	0 (0)	0	1 (6.7) ^d^	1.2–29.8	0 (0)	0

**Table 5:** Number and percentage of *Ixodes ricinus* positive for *Borrelia burgdorferi* s.l. species at individual study sites in Žemberovce (sites 3–6) and Horšianska Dolina National Nature Reserve (site 7). No. of positive/tested ticks (%), N—nymph, A—adult.

Study Sites	Stage	*Borrelia burgdorferi* s.s.	*Borrelia afzelii*	*Borrelia garinii*	*Borrelia valaisiana*
Site 3	N	0	1/3 (33.3)	0	0
	A	0	0	0	1/3 (33.3)
Site 4	N	0	2/19 (10.5)	1/19 (5.3)	1/19 (5.3)
	A	0	0	0	0
Site 5	N	0	3/16 (18.8)	0	0
	A	0	2/6 (33.3)	0	1/6 (16.7)
Site 6	N	0	4/20 (20)	1/20 (5)	1/20 (5)
	A	0	0	0	0
Site 7	N	1/58 (1.7)	8/58 (13.8)	5/58 (8.6)	0
	A	0	0	3/12 (25)	2/12 (16.7)

**Table 6:** Numbers and species of ticks obtained from game animals in Žemberovce hunting district. F—female, M—male, and N—nymph.

Tick Species	Stage	Hosts			Total (%)
		*S. scrofa*	*C. elaphus*	*C. capreolus*	
*I. ricinus*	F	3	48	5	56 (26.5)
	M	0	17	2	19 (9)
	N	30	1	0	31 (14.7)
*H. concinna*	F	5	6	0	11 (5.2)
	M	0	2	0	2 (0.9)
	N	7	1	0	8 (3.8)
*H. inermis*	F	10	3	0	13 (6.2)
	M	9	0	0	9 (4.3)
*D. reticulatus*	F	22	0	0	22 (10.4)
	M	11	0	0	11 (5.2)
*D. marginatus*	F	15	0	0	15 (7.1)
	M	14	0	0	14 (6.6)
Total (%)		126 (59.7)	78 (37)	7 (3.3)	211 (100)

**Table 7:** Number of positive engorged ticks and prevalence rates (%) with 95% confidence intervals by pathogen, tick species, developmental stage and animal host. A—adult, N—nymph, and *n*—number. ^a^ *Babesia microti*, ^b^ *Babesia crassa*, ^c^ *Babesia* sp. GNT1, ^d^ *Theileria* sp., ^e^ *Babesia* sp. GNT2, ^rh^ *Rickettsia helvetica*, and ^rr^ *Ricketsia raoultii*.

Host	Tick Species	Stage	Total	*Babesia*/*Theileria* spp.		*Rickettsia* spp.		*A. phagocytophilum*		*B. miyamotoi*	
			Tested	Positive *n* (%)	95% CI	Positive *n* (%)	95% CI	Positive *n* (%)	95% CI	Positive *n* (%)	95% CI
*S. scrofa*	*I. ricinus*	A	3	0 (0)	0	0 (0)	0	1 (33.3)	6.2–79.2	1 (33.3)	6.2–79.2
		N	30	2 (6.7) ^a^	1.9–21.3	2 (6.7) ^rh^	1.9–21.3	11 (36.7)	21.9–54.5	1 (3.3)	0.6–16.7
	*H. concinna*	A	5	2 (40) ^b,c^	11.8–76.9	0 (0)	0	2 (40)	11.8–76.9	0 (0)	0
		N	7	1 (14.3) ^c^	2.6–51.3	0 (0)	0	0 (0)	0	0 (0)	0
	*H. inermis*	A	19	0 (0)	0	1 (5.3) ^rh^	0.9–24.6	2 (10.5)	2.9–31.4	0 (0)	0
	*D. reticulatus*	A	33	1 (3.1) ^c^	0.5–15.3	3 (9.1) ^rr^	3.1–23.6	6 (18.2)	8.6–34.4	0 (0)	0
	*D. marginatus*	A	29	0 (0)	0	0 (0)	0	5 (17.2)	7.6–34.6	0 (0)	0
	Total		126	6 (4.8)	2.2–10.0	6 (4.8)	2.2–10.0	27 (21.4)	15.2–29.3	2 (1.6)	0.4–5.6
*C. elaphus*	*I. ricinus*	A	65	23 (35.4) ^d^	24.9–47.5	4 (6.2) ^rh^	2.4–14.8	54 (83.1)	72.2–90.3	0 (0)	0
		N	1	0 (0)	0	0 (0)	0	1 (100)	20.7–100	0 (0)	0
	*H. concinna*	A	8	3 (37.5) ^d^	13.7–69.4	0 (0)	0	5 (62.5)	30.6–86.3	0 (0)	0
		N	1	1 (100) ^e^	20.7–100	0 (0)	0	1 (100)	20.7–100	0 (0)	0
	*H. inermis*	A	3	2 (66.7) ^d^	20.8–93.9	0 (0)	0	2 (66.7)	20.8–93.9	0 (0)	0
	Total		78	29 (37.2)	27.3–48.3	4 (5.1)	2.0–12.5	63 (80.8)	70.7–88.0	0 (0)	0
*C. capreolus*	*I. ricinus*	A	7	0 (0)	0	0 (0)	0	1 (14.3)	2.6–51.3	0 (0)	0
Total	*I. ricinus*	A	75	23 (30.7)	21.4–41.9	4 (5.3)	2.1–12.9	56 (74.7)	63.8–83.1	1 (1.3)	0.2–7.2
		N	31	2 (6.5)	1.8–20.7	2 (6.5)	1.8–20.7	12 (38.8)	23.8–56.2	1 (3.2)	0.6–16.2
	*H. concinna*	A	13	6 (46.2)	23.2–70.9	0 (0)	0	7 (53.9)	29.1–76.8	0 (0)	0
		N	8	2 (25)	7.2–59.1	0 (0)	0	1 (12.5)	2.2–47.1	0 (0)	0
	*H. inermis*	A	22	2 (9.1)	2.5–27.8	1 (4.6)	0.8–21.8	4 (18.2)	7.3–38.5	0 (0)	0
	*D. reticulatus*	A	33	1 (3.1)	0.5–15.3	3 (9.1)	3.1–23.6	6 (18.2)	8.6–34.4	0 (0)	0
	*D. marginatus*	A	29	0 (0)	0	0 (0)	0	5 (17.2)	7.6–34.6	0 (0)	0
	Total		211	35 (16.6)	12.2–22.2	10 (4.7)	2.6–8.5	91 (43.1)	36.6–49.9	2 (1)	0.3–3.4

**Table 8:** Prevalence of pathogens detected in spleen samples of game animals in Žemberovce hunting district.

Host	Pathogen	Positive/Tested	Prevalence (%)
*Sus scrofa*	*Anaplasma phagocytophilum*	10/18	55.6
*Cervus elaphus*	*Anaplasma phagocytophilum*	13/13	100
	*Theileria capreoli*	9/13	69.2
	*Theileria* spp.	3/13	23.1
*Capreolus capreolus*	*Anaplasma phagocytophilum*	2/2	100
	*Theileria capreoli*	2/2	100

**Table 9:** Prevalence of pathogens (%) with 95% confidence intervals in ticks removed from sheep. *n*—number.

Tick Species	Total	*A. phagocytophilum*		*A. ovis*		*Rickettsia* spp.	
	Tested	Positive *n* (%)	95% CI	Positive *n* (%)	95% CI	Positive *n* (%)	95% CI
*Ixodes ricinus*	5	2 (40)	11.8–76.9	0 (0)	0	0 (0)	0
*Dermacentor marginatus*	42	2 (4.8)	1.3–15.8	2 (4.8)	1.3–15.8	4 (9.5)	3.8–22.1
*Haemaphysalis concinna*	1	0 (0)	0	0 (0)	0	0 (0)	0
Total	48	4 (8.3)	3.3–19.6	2 (4.2)	1.2–14.0	4 (8.6)	3.3–19.6

## Data Availability

All data generated and analyzed during this study are included in the published article, its Supplementary Materials, and online resource https://doi.org/10.6084/m9.figshare.31869478. Selected gene sequences are deposited in GenBank under accession numbers PX992076, PX991675-PX991680, and PZ171410-PZ171444.

## Supplementary Materials

The following supporting information can be downloaded at: https://www.mdpi.com/article/10.3390/pathogens15040382/s1, Supplementary File S1: Methods used for molecular detection of microorganisms; Supplementary Table S1: List of variants of the amplified gene sequences of *Borrelia burgdorferi* s.l. species, *Rickettsia* species, *Anaplasma phagocytophilum*, *Anaplasma ovis*, *Babesia* species and *Theileria capreoli*, designation of samples, GenBank accession numbers for submitted sequences, their sources, and numbers of identical sequences.
