# Supplementary material for: Sympatric Occurrence of Five Exophilic Tick Species in the Levice Region (Southwestern Slovakia) and Their Infection with Tick-Borne Pathogens
Source: Pathogens. 2026 Apr 2;15(4):382. doi: 10.3390/pathogens15040382 (PMC13119089; doi:10.3390/pathogens15040382)
Supplement: Supplementary file 1 [file pathogens-15-00382-s001.zip › Supplementary File S1.pdf]

Supplementary File S1. Methods used for molecular detection of microorganisms

Real-time PCR was carried out using a C1000 Touch™ Thermal Cycler with CFX96TM Real-Time System (Bio-Rad, CA). PCR amplifications were performed in a T100 Thermal cycler and C1000 Touch™ Thermal Cycler (both Bio-Rad, USA).

Anaplasma phagocytophilum DNA was detected using a probe-based real-time PCR with primer sequences specific for the *A. phagocytophilum msp*2 gene ApMSP2f and ApMSP2r to generate a 77-bp fragment by using a TaqMan probe identified as ApMSP2p-FAM (Courtney et al. 2004). The reaction mixtures of 25 µl contained: 12.5 µl 2x SuperHot Master mix (SuperHotTaq Polymease, PCR buffer, dNTPs and $MgCl_2$ optimized by Bioron GmbH, Germany), 0.625 µl $MgCl_2$ (100 mM), 1.8 µl forward and reverse primers (10 µM each), 0.5 µl probe (10 µM), 2.775 µl of nuclease-free H_2O and 5 µl of DNA template (unknown sample and positive control) or nuclease-free H_2O (negative control). The real-time PCR program consisted of three steps: 94 °C for 2 min, followed by 39 cycles at 95 °C for 15 s and 60 °C for 1 min. Samples were considered positive with an exponential rise of the curve and a ct-value (threshold cycle) <37.5.

Anaplasma phagocytophilum- positive samples were analyzed by nested PCR to identify *A. phagocytophilum* genotypes. Primers MSP4Ap5f and MSP4Ap3r in the first round and msp4f and msp4r in the second round, were used to amplify a 849-bp fragment of the *msp*4 gene (Pangrácová et al. 2013).

For the first round PCR, the 25 µl reaction mixtures contained 2.5 µl 10 × PCR Buffer, 1 µl $MgCl_2$ (25 mM), 0.5 µl 10 × dNTPs (10 mM), 0.5 µl forward and reverse primers (10 µM each), 0.125 µl HotstartTaq DNA polymerase (Qiagen, Hilden, Germany), 14.875 µl nuclease-free H_2O and 5 µl of DNA template (unknown sample and positive control) or nuclease-free H_2O (negative control). The first thermal cycle reaction consisted of an initial denaturation step at 95 °C for 5 min, followed by 40 cycles of denaturation at 94 °C for 30 s, annealing at 58 °C for 20 s and elongation at 72 °C for 1 min. A final extended amplification step at 72 °C for 5 min was carried out.

For the second round PCR, the 25 µl reaction mixtures contained 2.5 µl 10 × PCR Buffer, 1 µl $MgCl_2$ (25 mM), 0.5 µl 10 × dNTPs (10 mM), 0.5 µl forward and reverse primers (10 µM each), 0.125 µl HotstartTaq DNA polymerase (Qiagen, Hilden, Germany), 14.875 µl nuclease-free H_2O, 2 µl Milli-Q water and 3 µl of PCR product from the first round or nuclease-free H_2O (negative control). The second thermal cycle reaction consisted of an initial denaturation step at 95 °C for 5 min, followed by 35 cycles of denaturation at 94 °C for 30 s,

annealing at 58 °C for 20 s and elongation for at 72 °C 1 min. A final extended amplification step at 72 °C of 5 min was carried out.

A. phagocytophilum- positive samples were further analysed using the touchdown PCR method. A 530 bp fragment of the *groEL* gene was amplified (Alberti et al. 2005). For each reaction, 5 µl of DNA sample and 20 µl of reaction mixture were added to 0.2 ml tubes. The reaction mixture consisted of 2.5 µl of PCR buffer, 1 µl of $MgCl_2$, 0.5 µl of forward and reverse primers, 0.5 µl of dNTPs, 0.125 µl of DNA polymerase, and 14.875 µl of H_2O. The PCR protocol followed the manufacturer's instructions for the HotStartTaq Plus DNA Polymerase kit (Qiagen, Germany). Thermal cycle reaction consisted of an initial denaturation step at 95 °C for 5 min, followed by 41 cycles of denaturation at 94 °C for 30 s, annealing at 57 °C for 30 s, and elongation for at 72 °C for 45 s. A final extended amplification step at 72 °C of 5 min was carried out.

All tick samples from sheep were analysed using the touchdown PCR method. A 854-bp fragment of the *msp4* gene was amplified (De la Fuente et al. 2007). For each reaction, 5 µl of DNA sample and 20 µl of reaction mixture were added to 0.2 ml tubes. The reaction mixture consisted of 2.5 µl of PCR buffer, 1 µl of $MgCl_2$, 0.5 µl of forward and reverse primers, 0.5 µl of dNTPs, 0.125 µl of DNA polymerase, and 14.875 µl of H_2O. The PCR protocol followed the manufacturer's instructions for the HotStartTaq Plus DNA Polymerase kit (Qiagen, Germany). Thermal cycle reaction consisted of an initila denaturation step at 95°C for 5 min, followed by 39 cycles of denaturation at 94 °C for 30 s, annealing at 60 °C for 30 s, and elongation for at 68 °C for 1 min. A final extended amplification step at 72 °C of 5 min was carried out.

Borrelia miyamotoi was detected by the amplification of a 1,256 bp fragment of the 16S rRNA gene using Bmp41f and Bmp41r primers and the corresponding dye-labeled probe identified as Bmp41 (Platonov et al. 2011). The real-time PCR mix of 25 µl contained 12.5 µl 2x SuperHot Master mix (SuperHotTaq Polymease, PCR buffer, dNTPs and $MgCl_2$ optimized by Bioron GmbH, Germany), 0.625 µl $MgCl_2$ (100 mM), 1.8 µl forward and reverse primers (10 µM each), 0.5 µl probe (10 µM), 2.775 µl of nuclease-free H_2O and 5 µl of DNA template (unknown sample and positive control) or nuclease-free H_2O (negative control). Real-time PCR program consisted of these steps: 95 °C for 2 min, followed by 10 cycles at 95 °C for 20 s, 67 °C for 50 s, 72 °C for 20 s followed by 95 °C for 20 s and 60 °C for 50 s. Samples were considered positive with an exponential rise of the curve and a ct-value (threshold cycle) <37.5.

The forward primer Bb23Sf and the reverse primer Bb23Sr were used for detection of *Borrelia burgdorferi* s.l. We amplified a 75-bp fragment of 23S rRNA gene with a TaqMan probe identified as Bb23Sp-FAM (Courtney et al. 2004). 12.5 µl 2x SuperHot Master mix

(SuperHotTaq Polymease, PCR buffer, dNTPs and $MgCl_2$ optimized by Bioron GmbH, Germany), 0.625 µl $MgCl_2$ (100 mM), 1.8 µl forward and reverse primers (10 µM each), 0.5 µl probe (10 µM), 2.775 µl of nuclease-free H_2O and 5 µl of DNA template (unknown sample and positive control) or nuclease-free H_2O (negative control). Real-time PCR program, consisted of three steps: 94 °C for 2 min., followed by 39 cycles at 94 °C for 15 s and 60 °C for 1 min. Samples were considered positive with an exponential rise of the curve and a ct-value (threshold cycle) <37.5.

PCR products positive for *B. burgdorferi* s.l,. were re-analyzed by conventional PCR according to the protocol by Derdaková et al. (2003). *Borrelia* genus specific IgSA and IgSB primers were used to amplify a 300 bp portion of the 5S-23S (rrfA-rrlB) rDNA intergenic spacer. For this PCR, the reaction mixtures of 25 µl contained: 2.5 µl 10 × PCR buffer, 1 µl $MgCl_2$ (25 mM), 0.5 µl 10 × dNTPs (10 mM), 0.5 µl forward and reverse primers (10 µM each), 0.125 µl Taq Hotstart DNA polymerase (Qiagen, Hilden, Germany), 14.875 µl of nuclease-free H_2O and 5 µl of DNA template (unknown sample and positive control) or nuclease-free H_2O (negative control). Each sample was subjected to a touch-down PCR program, consisting of 11 steps: initial denaturation at 95 °C for 15 min., followed by 5 cycles at 94 °C for 15 s, 61 °C for 25 s (-0.2°C /cycle) and 72 °C for 30 s, followed by 5 cycles at 94 °C for 15 s, 60 °C for 25 s, at 72 °C for 30 s and completed by 30 cycles at 94 °C for 15 s, 58 °C for 25 s, at 72 °C for 30 s followed by last step at 72 °C for 30 s.

Babesia spp. DNA amplification by PCR was carried out following the protocol by Casati et al. (2006). *Babesia* genus-specific BJ1 and BN2 primers were used to amplify a 450 bp region of the 18S rRNA gene. PCR reactions were carried out in a volume of 25 µl PCR mix containing 2.5 µl 10 × PCR buffer, 1 µl $MgCl_2$ (25 mM), 0.5 µl 10 × dNTPs (10 mM), 0.5 µl forward and reverse primers (10 µM each), 0.125 µl Taq Hotstart DNA polymerase (Qiagen, Hilden, Germany), 14.875 µl of nuclease-free H_2O and 5 µl of DNA template (unknown sample and positive control) or nuclease-free H_2O (negative control). The thermal cycle reaction consisted of an initial step at 95 °C for 15 min, followed by 35 cycles at 94 °C for 1 min., 55 °C for 1 min. and 72 °C at 2 min. A final extended amplification step of 10 min at 72 °C was carried out.

Molecular detection of *Rickettsia* spp. was performed with primers D767f and D1390r amplifying a 626-bp fragment of the *sca4* gene (Sekeyova et al. 2001). All amplifications were performed in a total volume of 25 µl of reaction mixture containing 2.5 µl 10 × PCR buffer, 1 µl $MgCl_2$ (25 mM), 0.5 µl 10 × dNTPs (10 mM), 0.5 µl forward and reverse primers (10 µM each), 0.125 µl Taq Hotstart DNA polymerase (Qiagen, Hilden, Germany), 14.875 µl of nuclease-free H_2O and 5 µl of DNA template (unknown sample and positive control) or nuclease-free H_2O

(negative control). The thermal cycle reaction consisted of an initial step at 95 °C for 15 min, followed by 35 cycles at 95 °C for 20 s., 53 °C for 30 s. and 60 °C for 2 min. A final extended amplification step of 5 min at 72 °C was carried out.

PCR products from all reactions were analyzed by electrophoresis in 1.5% agarose gel, stained with GoodView Nucleic Acid Stain (Beijing SBS Genetech, Beijing, China) and visualized with an UV transilluminator. Positive PCR products were purified and sent for sequencing to Eurofins Genomics (Germany).

References

Casati, S.; Sager, H.; Gern, L.; Piffaretti, J.C. Presence of potentially pathogenic *Babesia* sp. for human in *Ixodes ricinus* in Switzerland. *Ann. Agric. Environ. Med*. **2006**; *13,* 65–70.

Courtney, J.W.; Kostelnik, L.M.; Zeidner, N.S.; Massung, R.F. Multiplex real-time PCR for detection of *Anaplasma phagocytophilum* and *Borrelia burgdorferi*. *J. Clin. Microbiol*. **2004**; *42,* 3164–8. doi:10.1128/jcm.42.7.3164-3168.2004.

Pangrácová, L.; Derdáková, M.; Pekárik, L.; Hviščová, I.; Víchová, B.; Stanko, M.; Hlavatá, H.; Peťko, B. *Ixodes ricinus* abundance and its infection with the tick-borne pathogens in urban and suburban areas of Eastern Slovakia. *Parasit. Vectors.* **2013;** *6*, 238. doi:10.1186/1756-3305-6-238

Alberti, A.; Zobba, R.; Chessa, B.; Addis, M.F.; Sparagano, O.; Parpaglia, M.L.P.; Cubeddu, T.; Pintori, G.; Pittau, M. Equine and canine *Anaplasma phagocytophilum* strains isolated on the Island of Sardinia (Italy) are phylogenetically related to pathogenic strains from the United States. *Appl. Eviron. Microbiol*. **2005**; *71,* 6418-6422. doi:10.1128/AEM.71.10.6418-6422.2005.

Sekeyova, Z.; Roux, V.; Raoult, D. Phylogeny of *Rickettsia* spp. inferred by comparing sequences of 'gene D', which encodes an intracytoplasmic protein. *Int J Syst Evol Microbiol.* **2001**; *51,* 1353–1360. doi:10.1099/00207713-51-4-1353

Derdáková, M.; Halanová, M.; Stanko, M.; Štefančíková, A.; Čisláková, L.; Pet'ko, B. Molecular evidence for *Anaplasma phagocytophilum* and *Borrelia burgdorferi* sensu lato in *Ixodes ricinus* ticks from eastern Slovakia. *Ann. Agric. Environ. Med*. **2003**; *10,* 269–271.

Platonov, A. E.; Karan, L.S.; Kolyasnikova, N.M.; Makhneva, N.A.; Toporkova, M.G.; Maleev, V.V.; Fish, D.; Krause, P.J. Humans infected with relapsing fever spirochete *Borrelia miyamotoi*, Russia. *Emerg. Infect. Dis*. **2011**; *17*, 1816. doi:10.3201/eid1710.101474

de la Fuente, J.; Atkinson, M.W.; Naranjo, V.; de Mera, I.G.F.; Mangold, A.J.; Keating, K.A.; Kocan, K.M. Sequence analysis of the msp4 gene of *Anaplasma ovis* strains. *Vet. Microbiol.* **2007**; *119*, 375-381. doi:10.1016/j.vetmic.2006.09.011
