# Supplementary material for: Sympatric Occurrence of Five Exophilic Tick Species in the Levice Region (Southwestern Slovakia) and Their Infection with Tick-Borne Pathogens
Source: Pathogens. 2026 Apr 2;15(4):382. doi: 10.3390/pathogens15040382 (PMC13119089; doi:10.3390/pathogens15040382)
Supplement: Supplementary file 1 [file pathogens-15-00382-s001.zip › Supplementary Table S1.pdf]

Table S1. List of variants of the amplified gene sequences of *Borrelia burgdorferi* s.l. species, *Rickettsia* species, *Anaplasma phagocytophilum*, *Anaplasma ovis*, *Babesia* species and *Theileria capreoli,* designation of samples, GenBank accession numbers for submitted sequences, their sources, and numbers of identical sequences.

**Target gene**<br>Pathogen<br>Sample designation	GenBank Access. No.	Questing tick species (No., stage)	Feeding tick species (No., stage) spleen (No.)	Host
***sca*4**				
Rickettsia raoulti GNT1	PX991675	-	*D. reticulatus* (2♀, 1♂)	*Sus scrofa*
Rickettsia raoulti GNT2	PX991676	*D. marginatus* (1♀)	*D. marginatus* (1♂)	*Ovis aries*
Rickettsia helvetica	PX991677	*I. ricinus* (1♀, 2♂, 4N)	*I. ricinus* (2♀, 2♂)<br>*I. ricinus* (2N)	*Cervus elaphus*<br>*Sus scrofa*
Rickettsia slovaca	PX991678		*D. marginatus* (2♀, 1♂)	*Ovis aries*
**18S rRNA**				
Babesia crassa	PX992076	*H. inermis* (1♂)	*H. concinna* (1♀)	*Sus scrofa*
Babesia microti		*I. ricinus* (1N)	*I. ricinus* (1N)	*Sus scrofa*
Babesia sp. GNT1		-	*H. concina* (1♀+1N)<br>*D. reticulatus* (1♂)	*Sus scrofa*<br>*Sus scrofa*
Babesia sp. GNT2			*H. concinna* (1♂)	*Cervus elaphus*
Theileria capreoli GNT1			spleen (1)	*Cervus elaphus*
			I. ricinus (2♀)	*Cervus elaphus*
Theileria capreoli GNT2			spleen (3)<br>spleen (2)	*Cervus elaphus*<br>*Capreolus capreolus*
groEL				
Anaplasma phagocytophilum				
S2	PZ171433		spleen	*Cervus elaphus*
S3	PZ171434		spleen	*Cervus elaphus*
S6	PZ171435		spleen	*Sus scrofa*
S10	PZ171436		spleen	*Sus scrofa*
S15	PZ171437		spleen	*Cervus elaphus*
S20	PZ171438		spleen	*Cervus elaphus*
S23	PZ171439		spleen	*Cervus elaphus*
S24	PZ171440		spleen	*Cervus elaphus*
S25	PZ171441		spleen	*Cervus elaphus*
S26	PZ171442		spleen	*Cervus elaphus*
S31	PZ171443		spleen	*Cervus elaphus*
S33	PZ171444		spleen	*Capreolus capreolus*
***msp*4**				
Anaplasma phagocytophilum				
S2	PZ171427		spleen	*Cervus elaphus*
S3	PZ171428		spleen	*Cervus elaphus*
S4	PZ171429		spleen	*Sus scrofa*
S10	PZ171430		spleen	*Sus scrofa*
S11	PZ171431		spleen	*Sus scrofa*
S22	PZ171432		spleen	*Capreolus capreolus*
Anaplasma ovis				
O5MI	PX991679		*D. marginatus* (1♂)	*Ovis aries*
O19F2	PX991680		*D. marginatus* (1♀)	*Ovis aries*
**5S-23S (rrfA-rrlB) rRNA**				
Borrelia afzelii GNT1	PZ171410	*I. ricinus* (5N)		

Borrelia afzelii GNT2	PZ171411	*I. ricinus* (1N)
Borrelia afzelii GNT3	PZ171412	*I. ricinus* (1N)
Borrelia afzelii GNT4	PZ171413	*I. ricinus* (2N, 1♂)
Borrelia afzelii GNT5	PZ171414	*I. ricinus* (1N)
Borrelia afzelii GNT6	PZ171415	*I. ricinus* (1N)
Borrelia afzelii GNT7	PZ171416	*I. ricinus* (4N)
Borrelia afzelii GNT8	PZ171417	*I. ricinus* (1N)
Borrelia afzelii GNT9	PZ171418	*I. ricinus* (1N)
Borrelia garinii GNT1	PZ171419	*I. ricinus* (2♀)
Borrelia garinii GNT2	PZ171420	*I. ricinus* (1N)
Borrelia garinii GNT3	PZ171421	*I. ricinus* (1♂)
Borrelia garinii GNT4	PZ171422	*I. ricinus* (2N, 1♀)
Borrelia valaisiana GNT1	PZ171423	*I. ricinus* (1N, 1♀)
Borrelia valaisiana GNT2	PZ171424	*I. ricinus* (1♂)
Borrelia valaisiana GNT3	PZ171425	*I. ricinus* (1♂)
Borrelia burgdorferi sensu stricto	PZ171426	*I. ricinus* (1N)
